# In-Depth Genomic and Transcriptomic Analysis of Five K^+^ Transporter Gene Families in Soybean Confirm Their Differential Expression for Nodulation

**DOI:** 10.3389/fpls.2017.00804

**Published:** 2017-05-23

**Authors:** Hafiz M. Rehman, Muhammad A. Nawaz, Zahid Hussain Shah, Ihsanullah Daur, Sadia Khatoon, Seung Hwan Yang, Gyuhwa Chung

**Affiliations:** ^1^Department of Biotechnology, Chonnam National UniversityYeosu, South Korea; ^2^Department of Arid Land Agriculture, King Abdul-Aziz UniversityJeddah, Saudi Arabia; ^3^Department of Biosciences, University of WahWah Cantt, Pakistan

**Keywords:** K^+^ transporters, HAK/KT/KUP, voltage-gated K^+^ channel, TPK/KCO, HKT, KEA, soybean

## Abstract

Plants have evolved a sophisticated network of K^+^ transport systems to regulate growth and development. Limited K^+^ resources are now forcing us to investigate how plant demand can be satisfied. To answer this complex question, we must understand the genomic and transcriptomic portfolio of K^+^ transporters in plants. Here, we have identified 70 putative K^+^ transporter genes from soybean, including 29 HAK/KT/KUP genes, 16 genes encoding voltage-gated K^+^ channels, 9 TPK/KCO genes, 4 HKT genes, and 12 KEA genes. To clarify the molecular evolution of each family in soybean, we analyzed their phylogeny, mode of duplication, exon structures and splice sites, and paralogs. Additionally, ortholog clustering and syntenic analysis across five other dicots further explored the evolution of these gene families and indicated that the soybean data is suitable as a model for all other legumes. Available microarray data sets from Genevestigator about nodulation was evaluated and further confirmed with the RNA sequencing data available by a web server. For each family, expression models were designed based on Transcripts Per Kilobase Million (TPM) values; the outcomes indicated differential expression linked to nodulation and confirmed the genes' putative roles. In-depth studies such as ours provides the basis for understanding K^+^ inventories in all other plants.

## Introduction

Potassium (K^+^) is the most widespread inorganic cation in plant cells, constituting up to 10% of plant dry matter, and plants have evolved a sophisticated network of K^+^ transport systems over millions of years (Leigh and Wyn Jones, 1984). Indeed, K^+^ deficiency symptoms reveal that the ion is vital to the regulation of plant growth and development, involved in many essential functions like protein biosynthesis, turgor-driven movement, osmoregulation, photosynthesis, and maintenance of plasma membrane potential. However, K^+^ resources have become limited, prompting an investigation of how to satisfy demand. This complex question can only be answered through understanding the genomic and transcriptomic portfolio of K^+^ transporters. Thus far, molecular knowledge of K^+^ transporters is available mainly from a few model species. This limited view on the dynamic involvement of K^+^ in diverse physiological processes can be misleading, but the massive data volume analyzed in modern approaches potentially address the problem. In this study, we therefore dissected all available genomic and transcriptomic data from wet-lab experiments and ran computational simulations to understand the five major K^+^-transport-related gene families in soybean (a model legume) and their role in nodulation. Moreover, we examined the transferability of our conclusions from model species to other plant species.

The five major K^+^ transporter families are as follows (Gomez-Porras et al., 2012; He et al., 2012): (1) the HAK/KT/KUP family of high-affinity transporters, (2) voltage-gated channels/shakers, (3) non-voltage-gated (tandem pore) TPK/KCO channels, (4) the HKT family of high-affinity transporters, and (5) the KEA family of efflux antiporters. Among them, the HAK/KT/KUP family is the largest and plays a critical role in plant growth and development, i.e., cell expansion, K^+^ acquisition and auxin distribution (Gierth et al., 2005; Gobert et al., 2007; He et al., 2012). To date, HAK/KT/KUP genes have been cloned from many plant species, including rice (Gupta et al., 2008; Yang et al., 2014; Chen et al., 2015), *Arabidopsis* (Gierth et al., 2005), tomato (Nieves-Cordones et al., 2007), pepper (Martinez-Cordero et al., 2004), lotus (Desbrosses et al., 2004), and grapevine (Davies et al., 2006). Respectively, 27, 13, and 31 HAK/KT/KUP genes have been identified in rice (Yang et al., 2009), *Arabidopsis* (Mäser et al., 2001), and poplar (He et al., 2012), based on genome-wide sequence analysis. Plant HAK/KT/KUP proteins possess 10–15 transmembrane domains (TMDs) with both the N- and the far longer C-termini on the intracellular side of the membrane (Gomez-Porras et al., 2012; Nieves-Cordones et al., 2016). This family exhibits considerable diversity in subcellular localization, ranging across the plasma membrane, tonoplast, and other endomembranes (Osakabe et al., 2013; Rigas et al., 2013). Furthermore, their expression patterns are diverse, observed in root meristems, vascular tissues, guard cells, fruits, and specialized organs such as flytraps (Osakabe et al., 2013; Scherzer et al., 2015). Functionally, some members contribute to root K^+^ uptake, notably through active K^+^ transport at low external concentrations (Nieves-Cordones et al., 2014).

Potassium channels from viruses to plants are characterized by a conserved amino acid motif (TVGYGD) in the narrowest stretch of the selectivity filter (Kuang et al., 2015). Three major categories of voltage-gated K^+^ channels exist in plants: (1) inward-rectifying (K_in_) channels (AKT1, KAT1, KAT2, and SPIK) mediating K^+^ uptake and activated by membrane hyperpolarization; (2) weakly-inward-rectifying (K_weak_) channels (AKT2/3) that mediate K^+^ uptake and release depending on local K electrochemical gradients; and (3) outward-rectifying (K_out_) channels (SKOR and GORK) mediating K^+^ release from the cell and activated by membrane depolarization (Shabala and Pottosin, 2010; Gomez-Porras et al., 2012). Voltage-gated K^+^ channels genes are expressed ubiquitously in various plant tissues, providing a possibility for the rapid K^+^ redistribution between plant parts and cellular compartments. TPK/KCO channels participates in pollen tube growth, stomatal closure, and radical development; five members have been found in *Arabidopsis* (Czempinski et al., 2002; Wang and Wu, 2013). The first molecular analysis of K^+^ channels in plants involved the identification of AKT1 (Sentenac et al., 1992) and KAT1 (Anderson et al., 1992), two *Arabidopsis* K^+^ channel genes. Recently, an OsAKT1 gene was functionally characterized in rice (Ahmad et al., 2016).

The plant HKT family has been extensively studied since the discovery of TaHKT2;1 from bread wheat (*Triticum aestivum*), a gene that encodes Na^+^ and K^+^ co-transportation and also preferred Na^+^-selective low-affinity Na^+^ transport in the presence of a millimolar Na^+^ in *Xenopus laevis* oocytes and yeast (Horie et al., 2009). This group has been split into at least two subfamilies based on analysis of their structure and transport properties across multiple plant species (Platten et al., 2006). Class I HKT transporters generally exhibits Na^+^ selective transport with poor K^+^ permeability (Horie et al., 2009). Recently, an OsHKT1;4 gene from rice (Suzuki et al., 2016) and two genes from soybean named GmHKT1 and GmHKT1;4 were functionally characterized as being linked with salt tolerance (Chen H. et al., 2011; Chen J. et al., 2011; Chen et al., 2014). HKT genes are ubiquitously expressed in root and shoots (Almeida et al., 2013; Nieves-Cordones et al., 2016).

KEA proteins belong to the superfamily of monovalent cation proton antiporters (CPA) and several of its members are involved in chloroplast-envelope K^+^ transport in *Arabidopsis* and rice (Aranda-Sicilia et al., 2012; Kunz et al., 2014; Sheng et al., 2014). In addition, KEA antiporter systems have been detected in chloroplast and mitochondrial membranes, as well as tonoplast and plasma membranes (Song et al., 2004). For more detailed information about these five K^+^ transporter gene families, the author would like to recommend the extensive review papers by Rodriguez-Navarro and Rubio (2006), Dreyer and Uozumi (2011) and Hamamoto et al. (2015).

Here, we took advantage of publicly available genome and transcriptome data to prepare an inventory of K^+^ transporters in soybean as a resource and model for other dicots. We focused especially on HAK/KT/KUP, HKT, voltage- and non-voltage-gated K^+^ channels, HKT and KEA gene families. In this study, we first identified all members from each family in soybean. We next elucidated the evolutionary relationships of these K^+^ transporters with functional genomics: analyses of phylogeny, gene structure, splice sites, mode of duplication, paralogs, and orthologs. We associated the resultant data with root hair nodulation using microarray and RNA-seq-based transcriptome profiling of all K^+^ transporters. These in-depth genomic and transcriptomic investigations will provide more knowledge of the K^+^-transporter physiological complex in soybean and should contribute to an understanding of similar systems in other crops.

## Methods

### Genome-wide search for K^+^ transporters

Putative K^+^ transporters were identified via screening with transporter-specific protein motifs in the conceptual proteome of *Glycine max* (Wm82.a2.v1). Screening was performed in the FUZZPRO program from EMBOSS (Rice et al., 2000; Gomez-Porras et al., 2012; Kuang et al., 2015), using the following 11 motifs. For HAK/KT/KUP genes: (1) [A,G] [D,S,G]-[V,L,I,M]-x-x[S,A]-P-L-Y; (2) [A,G]-[N,D,H,S]-[D,N]-x-G-[E,Q,D,N]-[A,G]; (3) [A,G,S]-[D,N] [G,S,A,C]-x-[L,I,V,F]-x-P-x-[V,I,L,M]-[A,S]; (4) G-[S,A,T,C]-E-[A,G]-x-[F,Y]-A-[D,N,E]-[L,I,V]-[G,C,S,A]x-F; (5) [Y,F]-x-x-x-x-x-[H,F,Y]-G-Y-x-[E,D]; for K^+^ channels: (6) [S,T]-x-xT-x-G-[Y,F,L]-G-[D,E], (7) R-[L,F]-x-R-[L,V,I,A,G]-x-[R,C,K][V,A,L,M], (8) [A,V,S]-Y-[L,I]-[I,L]-G-[N,I]-[M,I]-T-[N,A]-L[V,I]; for HKTs: (9) [S,T,A]-x-[F,Y,V,L,C]-x-[D,N,S]G, (10) [G,A]-[Y,F]-[G,A]-x-[V,A,I]-G-[L,M,Y,F]-[S,T]; and for KEA: (11) (G-x-G-x-x-G-x(n)-[DE].

New Motifs were also scanned with the MEME Suite web application (http://meme-suite.org/). Additionally, results were checked against BLASTP 2.2.28+ searches in the soybean genome, using known *Arabidopsis* and rice transporters of different classes as templates. To eliminate false-positives, the resultant raw data were curated semi-automatically. Sequences were discarded if they were <70% of the average length among the outermost motifs in corresponding *Arabidopsis* transporters. All genomic annotation data (e.g., chromosomal location, definition, and annotation) were obtained from Phytozome https://phytozome.jgi.doe.gov/pz/portal.html. The relative lengths and positions of all available domains in the five K^+^ transporter families were graphically depicted using information obtained with the Simple Modular Architecture Research Tool (SMART, http://smart.embl-heidelberg.de/). Data on protein molecular weight (kDa) (http://web.expasy.org/compute_pi/) and TMDs (http://hmmer.org) were also collected.

### Phylogenetic analyses of K^+^ transporters in soybean

To gain insight into the evolution of soybean K^+^ transporter families, the reference gene sequences were obtained from NCBI database by using their gene IDs (Gomez-Porras et al., 2012). Multiple amino-acid sequence alignments of K^+^ transporters were generated in ClustalW with the default settings. The bootstrap consensus (1,000 replicates) trees were constructed using theMaximum Likelihood using RAxML and 1,000 bootstrap replicates (Stamatakis, 2006).

### Chromosomal location and expansion patterns

To categorize K^+^ transporter gene family expansion, we examined the chromosomal locations of all members in soybean (JGI v1.0) and generated a map in MapChart. Segmental duplication, tandem duplication, and transposition events cause gene family expansion (He et al., 2012). In this study, we focused on segmental and tandem duplication. Paralogous segments were created through two whole-genome duplication events in the soybean, 59 and 13 million years ago (Schmutz et al., 2010). Segmental duplications were identified following He et al. (2012). The Ks values of duplicated genes are similar over time assuming a molecular clock (Shiu et al., 2004); hence, to estimate dates of segmental-duplication events, Ks values were cast off and their means calculated for each gene pair inside a duplicated block. The estimated date of the duplication event was then predicted with the mean Ks (T = Ks/2λ) values and assuming clock-like rates (λ) of 1.5 × 10^−8^ identical substitutions/synonymous site/year for 6.1 × 10^−9^ (Lynch and Conery, 2000). Syntenic relationships were determined among all paralogs in Circoletto (http://tools.bat.infspire.org/circoletto/) to check the pattern of evolution.

### Genes structure analysis

Intron maps were constructed in accordance with a previously established method (Barvkar et al., 2012). Introns were classified based on their phase, length, and number in the genome. Diagrams of gene structures were drawn in Gene Structure Display Server (GSDS) (http://gsds.cbi.pku.edu.cn/). Exon boundaries within the coding regions of each tandem- or segmentally duplicated pairs were determined according to the thirty-first release of the Gramene database (http://www.gramene.org/). The nucleotide numbers (nt) for each exon as well as the phase of each splicing site were also determined. The potential domains of each K^+^ transporter family member were identified based on the Pfam database (He et al., 2012) to better understand its evolution within families.

### Ortholog identification and clustering pattern

Putative K^+^ transporters were searched against the NCBI Non-Redundant BLAST database with Blast2Go (*E* = 0.001) to identify orthologs in the genomic sequences of five dicots (*Phaseolus vulgaris, Arabidopsis thaliana, Vitis vinifera, Populus trichocarpa*, and *Medicago truncatula*), selected based on genome homologies. Sequences were considered orthologous at >80% similarity and their accession numbers were recorded. Orthologous peptide sequences were downloaded to investigate the clustering pattern of K^+^ transporter families in other genomes. Clustering analysis was performed in Orthovenn (Wang et al., 2015), and Venn gene clusters were generated for each family.

### Differential expression of K^+^ transporters in soybean

Microarray data of putative K^+^ transporters, obtained from the Genevestigator database (https://www.genevestigator.com/gv/), were used to detect expression in five developmental stages (flowering, fruit formation, germination, bean development, and main shoot growth) and 68 anatomical parts. Data collection focused on probe set used, developmental stage, and tissues in which putative genes were expressed. A heatmap was generated following published methods (Rehman et al., 2016).

Microarray data were further analyzed to clarify the possible roles of K^+^ transporters during nodulation. Although K^+^ transporters are widely expressed throughout plant tissues, this potential link to nodulation was deduced based on reports of LjKUP gene induction during nodule development in *Lotus japonicas* (Desbrosses et al., 2004) and the involvement of sulfate and histidine transporters in soybean nodulation (Libault et al., 2010). Perturbation analysis of the obtained microarray data was also performed using Genevestigator tool to generate heatmap on the basis of *Bradyrhizobium-japonicum*-treated and mock-treated William-82 root hair samples and the expression was examined at 6, 12, 18, 24, and 48 HAI (hours after inoculation).

### RNA-seq-based model development for nodulation

Nodulation-related differential expression of all K^+^ transporters was further confirmed with RNA-sequence data (Libault et al., 2010). The 78,773 soybean gene models were filtered with the RNA-seq browsing tool (http://118.178.236.158/SoyFN/). TPM values of zero to maximum in at least one of the seven stages (Root Hair 12HAI, Root hair_12HAImock, Root Hair 24 HAI, Root hair_24HAImock, Root Hair 48 HAI, Root hair_48HAImock) were calculated for all putative genes. On the basis of TPM values models were developed for each family and their responsible clades.

## Results

### Identification and phylogenetic analyses

Our screenings putatively identified 29 HAK/KT/KUP genes, 16 genes encoding voltage-gated K^+^ channels, 9 genes encoding TPK/KCO channels, 4 HKT genes, and 12 KEA genes (Table S1). Together with previously reported sequences, 70 putative genes are possibly involved with K^+^ transport in soybean (Table S1). Phylogenetic trees were constructed from proteins with motifs and domains selected for screening (Figures S1, S2).

**Figure 1:**
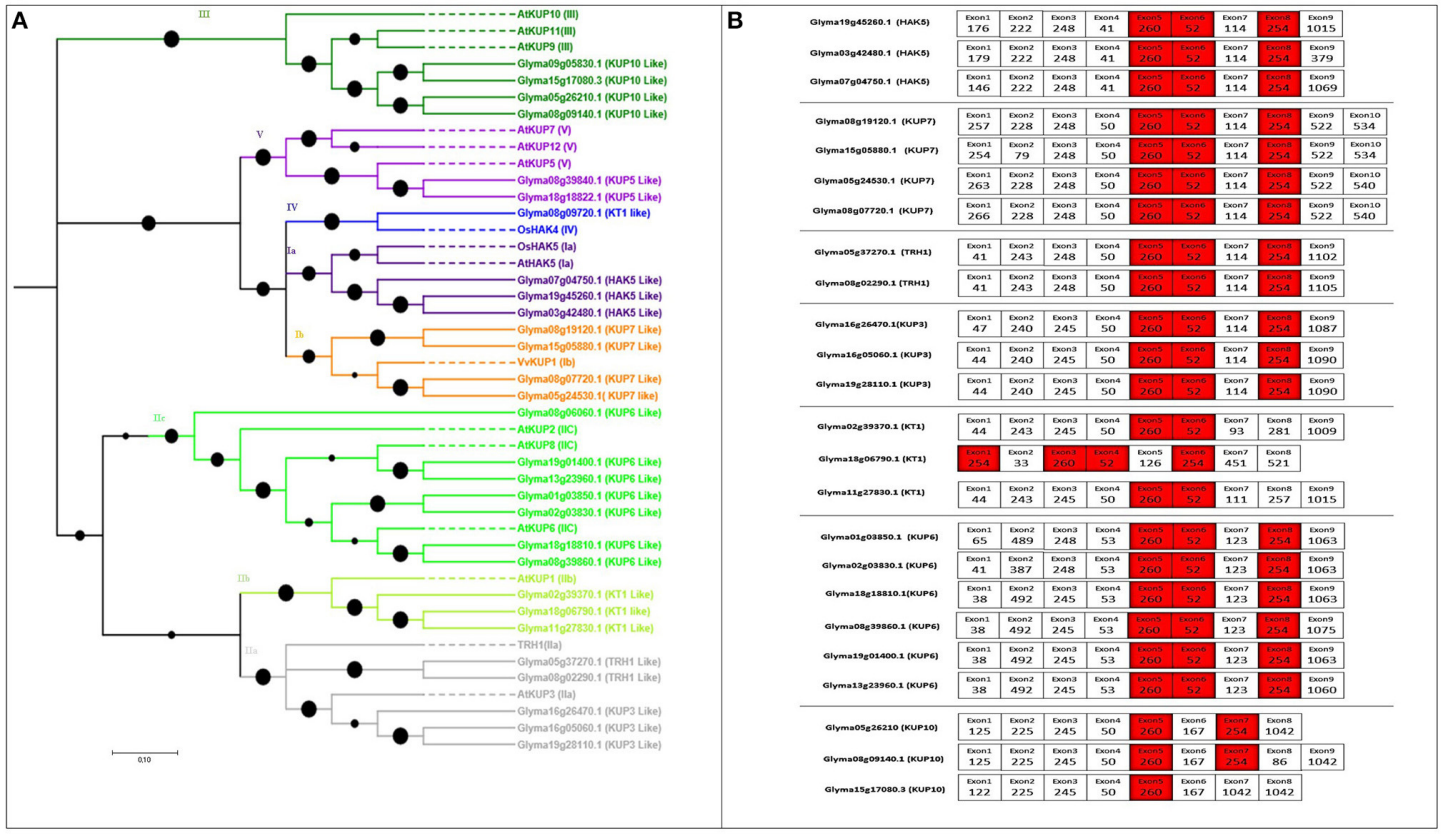
**Phylogenetic tree of putative HAK/KT/KUP transporters in soybean with their exon structure and splice sites. (A)** Five clearly distinguished clades of HAK transporters are present in soybean. Each represents an independent group of paralogs. To construct the phylogenetic tree, reference genes were taken from *Arabidopsis*, rice, and grapes (indicated as dotted lines). Soybean genes were shown by bold lines **(B)** Exon structure (5′–3′) and splice-site analysis of each tandemly or segmentally duplicated soybean HAK/KT/KUP pair. Nucleotide lengths are given as numbers in boxes. Exons colored red are conserved in length among all soybean HAK/KT/KUP genes. Exons colored white are conserved in length between each duplicated pair. Black colored filled circles on the phylogenic tree are showing branch lengths.

The number of genes identified in soybean (29) was greater than in rice (27) or *Arabidopsis* (13), and the same as in poplar (29). The length of HAK transporters in soybean ranged from 345 to 847 aa, and the exon number ranged from 1 to 10, with 12–14 TMDs (Table S1). The phylogenetic tree was constructed with 12 *Arabidopsis* genes, two rice genes, and one grape gene following previously described methods (Nieves-Cordones et al., 2016); five major clades were observed (I to V; Figure 1A), where I to IV followed previous numerations (Gomez-Porras et al., 2012; Very et al., 2014). Several subgroups were identified in clade I (Ia and Ib) and in clade II (IIa, IIb, and IIc) (Gupta et al., 2008; Nieves-Cordones et al., 2016). The HAK genes from each of the groups were found in all studied species of higher plants except *Arabidopsis*, where IV group is missing. Groups IIa and IIc constituted the largest clades, respectively containing five and seven soybean HAKs. Additionally, groups Ia and Ib clustered to form a larger clade, implying that they split from a common ancestor through subsequent gene duplications. The same clustering was observed for groups IIa and IIb.

**Figure 2:**
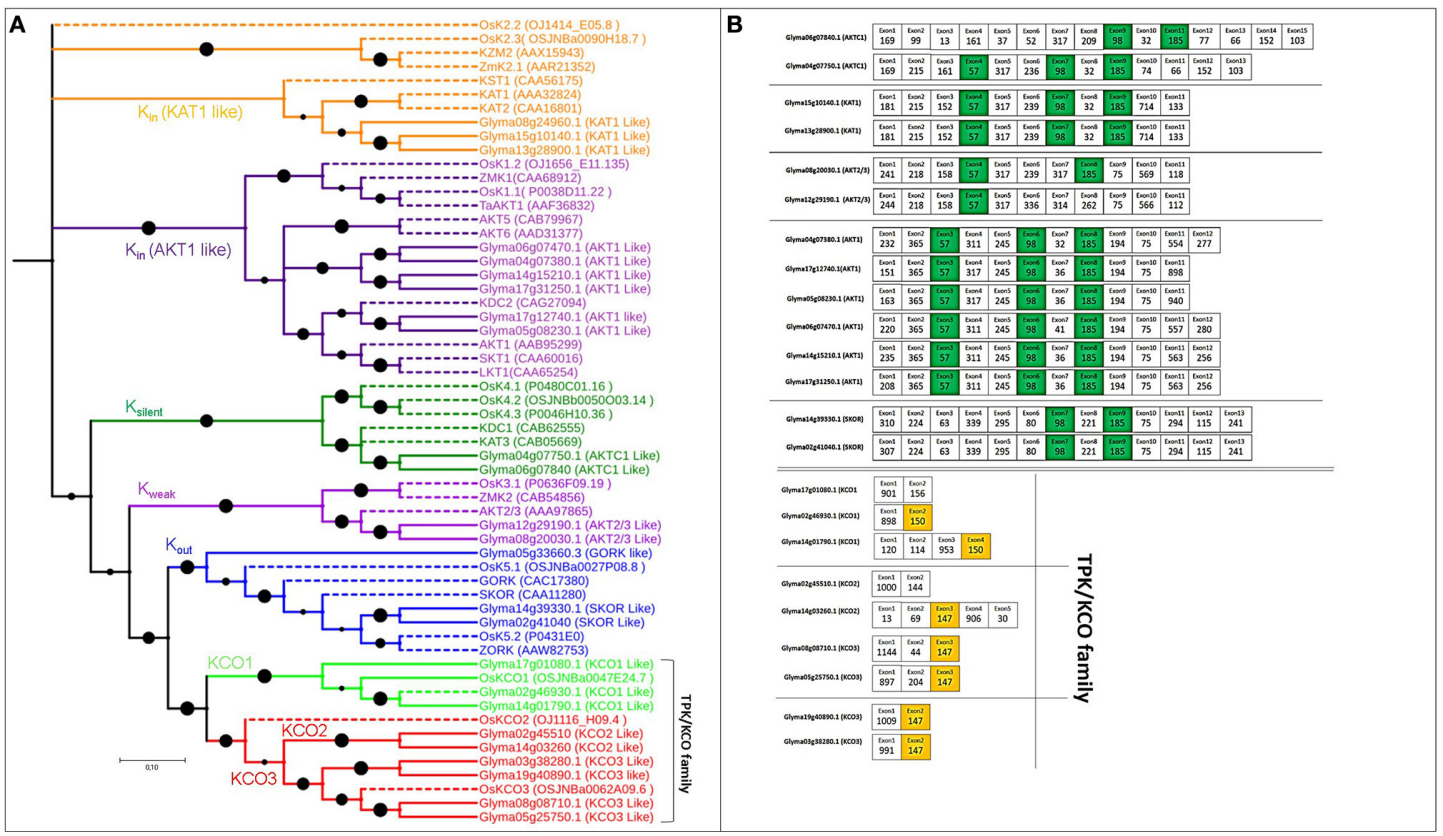
**Phylogenetic tree of putative Voltage-gated K^+^ channel and TPK/KCO genes in soybean, with exon structure and splice site analysis**. **(A)** Evolutionary relationships among genes encoding voltage-gated and TPK/KCO channels. Extensive functional analysis identified K_in_, K_out_, K_weak_, and K_silent_ channel subunits in the voltage-gated family that modulates K^+^ uptake. The TPK/KCO family clearly splits into two main groups, shown in bright green and red. Reference genes for constructing the phylogenetic tree are indicated in dotted lines with their NCBI gene names. **(B)** Exons colored green and orange are conserved in length among all soybean voltage-gated and TPK/KCO channels, respectively.

The 16 voltage-gated K^+^ channel genes identified based on pore-forming regions were greater in number than orthologous *Arabidopsis* (9) or poplar genes (11). The length of these genes in soybean ranged from 424 to 879 aa, and the exon number ranged from 11 to 15, with 4–6 TMDs (Table S1). Based on established methods (Gomez-Porras et al., 2012), we divided the phylogenetic tree of voltage-gated K^+^ channel genes into four functional subgroups: (a) nine K_in_ genes, (b) two K_silent_ genes, (c) two K_weak_ genes, (d) three K_out_ genes (Table S1 and Figure 2A).

Nine genes coding for TPK/KCO channel subunits were identified, a count that was greater than the TPK genes in *Arabidopsis* (6) and rice (3), but lower than poplar genes (10). Gene lengths in soybean ranged from 352 to 446 aa, and the exon number ranged from 2 to 5, with 4–5 TMDs (Table S1). The phylogenetic tree indicated that the soybean genes were divided into two clear groups, one containing the three KCO1 genes and the other with two and four genes from KCO2 and KCO3, respectively (Figure 2A).

**Figure 3:**
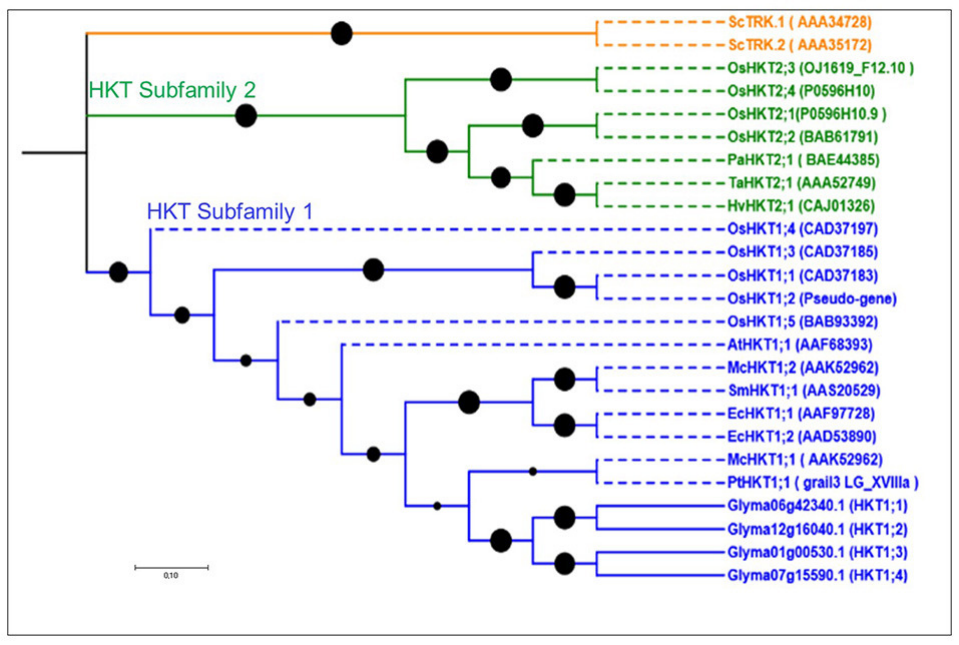
**Phylogenetic tree of putative HKT genes in soybean**. This family is represented by two subfamilies shown in green and blue. All soybean genes belong to HKT subfamily 1. Reference genes are shown in dotted lines with their NCBI gene names. No common splice site was found in the four soybean HKT genes.

Our screenings identified four HKT putative genes in soybean, more than in *Arabidopsis* (1) and poplar (1), but fewer than in rice (7). Gene lengths in soybean ranged from 410 to 574 aa, and the exon number ranged from 2 to 3, with 4–10 TMDs (Table S1). Phylogenetic analyses grouped all soybean HKTs into one group (Figure 3), indicating that the most recent common ancestor of these plants contained a single HKT-type protein. All four genes in soybean were part of the HKT subfamily 1 (Figure 3).

**Figure 4:**
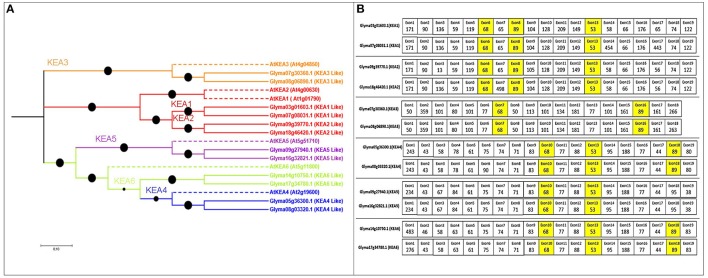
**Phylogenetic tree of the putative KEA family in soybean, with exon structure and splice site analysis. (A)** Three clearly distinguished clades are present in soybean. Referenced genes are indicated with dotted lines with their NCBI names. **(B)** Exons colored yellow are conserved in length among all soybean KEA genes.

Twelve putative KEA genes in soybean (more than the six in *Arabidopsis*) exhibited high percentage identity (Table S1). The exon number in the KEA gene family ranged from 19 to 21, coding for 576–1206 aa, with 9–12 TMDs. The phylogenetic tree revealed that the soybean KEA family split into three major clades: I (KEA3), II (KEA2 and KEA1), and III (KEA4, KEA5, and KEA6) (Figure 4A).

### Chromosomal location and expansion pattern

**Figure 5:**
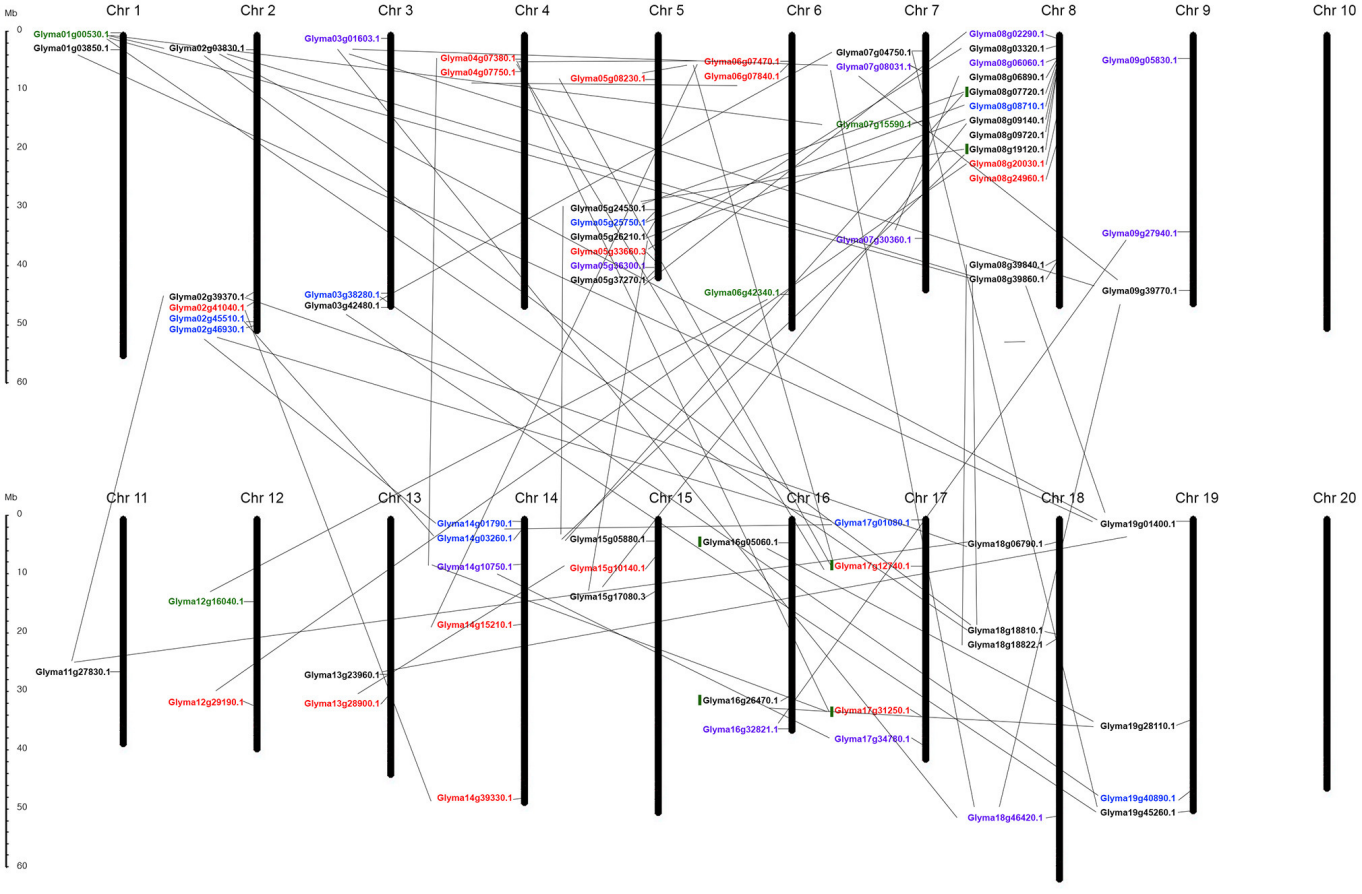
**Chromosomal distribution of all 70-putative K^+^ transporter genes and their duplication within genome**. All chromosomes have K^+^ transporter related genes except Chr 10 and Chr 20. Black, red, blue, green, purple indicate genes belonging to HAK/KT/KUP, voltage-gated K^+^ channel, TPK/KCO, HKT, and KEA families respectively. Gray lines represent gene duplications in each chromosome.

K^+^-transporter-related genes were unevenly distributed in the soybean genome, located in 18 of the 20 chromosomes, with chromosome 8 containing the highest number (Figure 5).

Chromosomes 4, 6, 10, 14, 17, and 20 harbored no HAK/KT/KUP genes (Figure 5), while relatively high densities of HAKs (eight genes), with some apparent tandem duplications, were discovered in chromosome 8; chromosomes 1, 3, 7, 9, 11, and 13 each possessed a single HAK gene (Figure 5). Based on phylogenetic clusters, two pairs of tandemly duplicated paralogs were discovered (Glyma08g19120.1/Glyma08g07720.1 [HAK7], Glyma16g26470.1/Glyma16g05060.1 [KUP3]). Of the 29 genes, only three were located outside duplicate blocks. The remaining HAK/KT/KUP genes were segmentally duplicated. Overall, this family experienced three rounds of whole genome duplication (WGD) events in the soybean. Most were duplicated around 59 and 13 million years ago (mya), except Glyma19g01400.1, which was segmentally duplicated 198 mya from Glyma01g03850.1 (Table S2). Furthermore, syntenic analysis among paralogs confirmed the mode of duplicated regions within the gene pairs (Figure S3).

The 16 AKT genes were also unevenly distributed, found only in chromosomes 2, 4, 5, 6, 8, 13, 14, and 17 (Figure 5). One pair of tandemly duplicated paralogs (Glyma17g12740.1/Glyma17g31250.1 [AKT1]) was discovered. Of the 16 genes, only two were located outside duplicate blocks. Segmental duplication was dominant during the four rounds of WGD in this family, occurring to all pairs except two. Some genes were duplicated around 70–256 mya, indicating that these genes were duplicated individually and later became the part of the soybean genome (Table S2). Syntenic analysis confirmed the conserved regions during duplication (Figure S3).

The nine putative KCO genes were distributed only on chromosomes 2, 3, 5, 8, 14, 17, and 19, with approximately one to two genes per chromosome (Figure 5). This family was entirely duplicated through segmental duplication, rather than tandem or miss duplication, under one round of WGD (13–59 mya). However, two genes (Glyma17g01080.1 and Glyma02g46930.1) were duplicated around 120 mya (Table S2). Syntenic analysis among paralogs confirmed the mode of duplication in this family (Figure S3).

All four putative HKT genes were segmentally duplicated and distributed only on chromosomes 1, 6, 7, and 12 (Table S2 and Figure 5). This family experienced one round of WGD around 16–28 mya. The conserved regions remained the same during duplication (Figure S3).

The 12 KEA genes were distributed only on chromosomes 3, 1, 7, 8, 9, 12, 14, 16, 17, and 18 (Figure 5). Six pairs of segmentally duplicated paralogs were discovered in this family (Table S2). The KEA1 and KEA2 gene pairs were duplicated through three rounds of WGD around 8–36 mya (Table S2). The remaining genes (KEA3, KEA4, KEA5, and KEA6) were segmentally duplicated around 6–8 mya, suggesting an evolution from KEA1 and KEA2 (Table S2). Syntenic analysis revealed similarities between the coding regions within each KEA gene pair (Figure S3).

### Gene structure and splice site analyses

Conserved exon structures (exons with the same nucleotide number and conserved intron phases) indicate similarities between genes (He et al., 2012; Tables S3, S4). Excluding those in HKT, a number of exons are conserved in length and possess nearly the same splice phases among HAK/KT/KUP, voltage-gated channels, TPK/KCO, and KEA families, respectively (Figures 1B, 2B, 4B). Intron phases were also highly conserved across all the segmentally and tandemly duplicated pairs of each family (Table S4 and Figure S4).

In the HAK/KT/KUP family, three exons (260, 52, and 254 nt) were highly conserved across all members except Glyma18g18822.1, Glyma02g39370.1, and Glyma11g27830.1, which do not have the 254-nt exon (Figure 1B). In addition to these three exons, each duplicated HAK pair shared several equal-length exons with the same splice phase. At least four conserved exon structures were shared by duplicated HAK pairs (Figure 1B).

All voltage-gated K^+^ channel genes possessed three highly conserved exons with lengths of 98, 57, and 185 nt, except Glyma08g20030.1 and Glyma12g29190.1, which lacked the 98-nt exon (green color in Figure 2B). Additionally, four to five exons of equal length, with the same splice and intron phases, were shared by each duplicated pair, again supporting the presence of a common ancestor (Figure 2B).

The TPK/KCO channel genes had two highly conserved, 150- and 147-nt exons (Figure 2B). Interestingly, no conserved exons were present across every member in this family, although each duplicated pair contained some conserved exons (Table S3). Intron phases remained conserved in this family, suggesting strong conservation throughout the family's evolutionary history (Table S4).

The KEA gene family also exhibited three highly conserved exons (68, 53, and 89 nt) (Figure 4B). The 53- and 89-nt exons were slightly variable among the duplicated KEA gene pairs. All gene pairs also contained 13–15 exons of equal length, with same splice site and intron phases (Tables S3, S4, and Figure 4B). The highly conserved exons support a common ancestor for the duplicated KEA genes.

### Ortholog identification and clustering pattern

In total, we identified 157 orthologs (>80% similarity) for 70 K^+^ transporter genes from five other legume proteomes (Table S5). Syntenic regions among the orthologs of five different families were identified in five separate dicots (Figure S5). Almost all annotated protein-coding genes in each family from these dicots were highly conserved (Figure S5).

We identified 66 orthologs with 2 clusters in the HAK/KT/KUP family, common across all five dicots (Figure S6). *Arabidopsis* had only one cluster for this family, suggesting that duplication expanded the HAK/KT/KUP family in the genomes of soybean and other dicots.

In voltage-gated K^+^ channel genes, a clustering pattern was observed among 33 identified orthologs, with one cluster in soybean and *P. vulgaris*, and two clusters in the other four dicots (Table S5 and Figure S6). This outcome implies that this family expanded in *Medicago truncatula, Vitis vinifera, Populus trichocarpa*, and *Arabidopsis* during evolution.

The remaining TPK/KCO, HKT, and KEA families had one cluster among all the orthologs, confirming their conservative mode of evolution through one common ancestor (Table S5 and Figure S6).

### Differential expression for nodulation

Of the 70 K^+^ transporter genes, we found 26 genes that were unevenly expressed across five developmental stages (germination, main shoot growth, flowering, fruit formation) and 68 anatomical parts (Figures S7–S10). Anatomical tissues were further subdivided into five sub groups: (a) primary cell, (b) seedling, (c) inflorescence, (d) shoot, and (f) roots. In the HAK/KT/KUP family, 12 genes were highly expressed at germination, main shoot growth, and bean development (Figure S7A), but lowly expressed during flowering and fruit formation. The 12 genes were highly expressed in primary cells (paraveinal mesophyll cells and palisade parenchyma cells), seedlings (maturation zone and root hair), inflorescence (androecium, stamen, anther, and pollen), and roots (stele, pericycle, and root tip) (Figure S7B), but lowly expressed in shoots. Microarray data for HAK/KT/KUP genes revealed that Glyma19g45260.1 (HAK5), Glyma19g01400.1 (KUP6), and Glyma9g05830.1 (KUP10) were highly expressed across the *B. japonicum*-mock-treated William 82 root hair cells at 6, 12, 18, 24,36, 48 HAI (Figure S7C).

Six voltage-gated K^+^ channel genes were highly expressed during main shoot growth, flowering, and fruit formation (Figure S8A). Glyma14g39330.1 (SKOR type) was 100% expressed at flowering. These genes were maximally expressed at inflorescence (flower, androecium, stamen, anther, pollen), shoots (leaf, trifoliate leaf), and roots (pericycle and nodule) (Figure S8B). Microarray data revealed that Glyma17g12740.1 (AKT1) and Ghlyma14g39330.1 were 20% expressed in the *B. japonicum*-mock-treated root hair cells at 6 and 14 HAI (Figure S8C).

In the TPK/KCO family, we found two genes actively expressed at germination, main shoot growth, fruit formation, and bean development (Figure S9A). Glyma02g46930.1 (KCO1) was maximally expressed at germination, while Glyma14g01790 (KCO1) was consistently expressed across all five developmental stages. These two genes were highly expressed in seedlings (radical, maturation zone, and root hair), roots (primary root, root tip, stele, and pericycle), and inflorescence (shoot apical meristem, parenchyma) (Figure S9B). Glyma02g46930.1 and Glyma14g01790 (KCO1) were highly expressed in root hair cells mock-treated with *B. japonicum* at 6, 12, 18, 24, 36, and 48 HAI (Figure S9C). Interestingly, we did not find any HKT genes from the microarray data analysis.

The six KEA genes were actively expressed during main shoot growth, fruit formation, and bean development (Figure S10A). Glyma08g03320.1 (KEA4) was consistently expressed at all five stages of development. Moreover, these genes were highly expressed in the primary cell stage (leaf, mesophyll, paraveinal, and palisade parenchyma cells), inflorescence (seed, embryo, shoot apical meristem, and root apical meristem), shoots (leaf and trifoliate leaves), and roots (root tip and nodule) (Figure S10B). Glyma17g34780 (KEA6) was actively expressed in root hair cells mock-treated with *B. japonicum* at 6 and 12 HAI (Figure S10C).

### RNA-seq based transcriptome profiling of K^+^ transporters in response to nodulation

**Figure 6:**
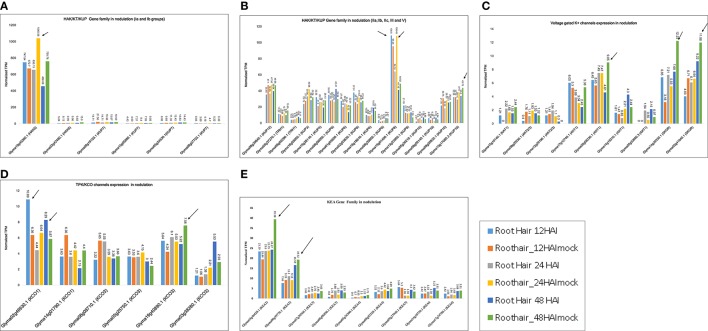
**K^+^ transporter gene expression models derived from RNA-seq data for nodulation**. Normalized TPM for all genes was recorded based on expression in *B. japonicum*-treated and mock-treated William 82 root hair samples at 6, 12, 18, 24, and 48 h after inoculation (HAI). Phylogeny-based models represent expression under at least one condition. **(A,B)** Clades 1a, Ib, IIa, IIb, IIc, III, and V genes from the HAK/KT/KUP family exhibit nodulation-related expression. **(C)** Expression of voltage-gated K^+^ channel genes in nodulation. **(D)** Expression of TPK/KCO genes in nodulation. **(E)** Expression of KEA family genes in nodulation. Arrows are showing the highest expression within a gene family.

The differential expression of K^+^ transporter families was further confirmed by RNA-seq data (Table S6). We found 22 HAK/KT/KUP family genes that had TPM values >1. In phylogenetic groups Ia and Ib, Glyma19g45260.1 (HAK5) was maximally expressed at 24 and 48 HAI to *B. japonicum*, with average TPM values of 1,040.58 and 759.8, respectively (Figure 6A). Generally, HAK5 and KUP7 exhibited maximum TPM values in mock-treated root hair cells. In groups IIa, IIb, and IIc, Glyma19g01400 (KUP8) was maximally expressed at 24 HAI, with an TPM value of 108.45 (Figure 6B). In group III, Glyma15g1780.3 was maximally expressed at 24 and 48 HAI, with TPM values of 37.9 and 43.78, respectively (Figure 6B). Group IV did not exhibit absolute expression for nodulation. In group V, Glyma08g39840.1 (KUP12) was expressed at 24 and 48 HAI, with TPM values of 47.58 and 46.56, respectively (Figure 6B).

Nine voltage-gated K^+^ channel genes exhibited medium-level expression for nodulation, ranging from 1 to 12 TPM. Two genes, Glyma14g39330.1 and Glyma02g41040.1 (K_*out*_, SKOR type), were more highly expressed, with TPM values of 12.23 and 11.98 at 48 HAI (Figure 6C). Another gene expressed at 48 HAI was Glyma05g08230.1 (K_*in*_, AKT1), with an TPM value of 9.05. These data reveal that voltage-gated K^+^ channel genes were mostly expressed at 48 HAI (Figure 6C).

Two TPK/KCO channel genes were expressed (TPM ~1–12) for nodulation; the highest expression for Glyma02g46930.1 (KCO1) and Glyma19g40890 (KCO3) occurred at 12 and 48 HAI (Figure 6D). Among the HKT family, Glyma12g16040.1 (HKT1;2) was the sole gene expressed only at 12 HAI (TPM = 4). In sum, this family was not found to be involved in soybean nodulation.

Nine KEA genes were dominantly expressed, with TPM values ranging from 1 to 45 (Figure 6E). Two genes, Glyma18g46420.1 (KEA2) and Glyma09g39770.1 (KEA2), were more highly expressed than the others at 48 HAI, with TPM values ranging from 39.38 to 19.32, respectively (Figure 6E). In general, KEA3, KEA4, and KEA6 were not expressed dominantly for nodulation.

## Discussion

### HAK/KT/KUP family in soybean

Although only a relatively small number of HAK/KT/KUP transporters have established physiological roles, our present analyses allow us to draw some major conclusions regarding phylogenetically based evolution, gene structure, expansion pattern, and differential expression (based on microarray and RNA-seq data). With 29 genes, the HAK/KT/KUP family in soybean is the same size as in poplar (Gomez-Porras et al., 2012; Nieves-Cordones et al., 2016). In this study, the HAK/KT/KUP phylogenetic tree strictly followed the same distribution into five groups, corroborating data from Nieves-Cordones et al. (2016) (Figure 1A). The maximum number of genes was distributed into Groups IIb and IIc. Like other soybean genes, Glyma19g45260.1, Glyma03g42480.1, and Glyma07g04750.1 (HAK5) are all involved in root high-affinity K^+^ uptake and fall into clade Ia (Martinez-Cordero et al., 2004; Nieves-Cordones et al., 2007, 2016; Rubio et al., 2008; Aleman et al., 2009; Gomez-Porras et al., 2012). In rice, clade Ia genes OsHAK5 and OsHAK21 exhibited a more specialized function of transporting K^+^ to aerial parts during K^+^ deficiency and salt stress, respectively (Yang et al., 2014; Shen et al., 2015). The soybean putative genes in the same clades (IIb and IIc) likely have the putative function of K^+^ transportation to aerial parts under salt stress and K^+^ deficiency.

As seen in data from other plants, clade Ib HAK/KT/KUP transporters may contribute to high-affinity K^+^ uptake in digesting glandes of Venus flytrap and flowers and berry skin of, (grapevine) (Davies et al., 2006; Scherzer et al., 2015; Nieves-Cordones et al., 2016). Further characterization of clade Ib transporters will clarify whether they are specialized in tissue or root K^+^ transport. Therefore, group Ib genes could be considered to determine their putative role in high K^+^ uptake during flowering (Figure 1A).

The *Arabidopsis* clade IIa (AtKUP4/TRH1) gene contributes to the polar localization of auxin transporters in the root apex, a necessary process for both gravitropic responses and root hair formation (Rigas et al., 2013; Nieves-Cordones et al., 2016). In clade IIc, AtKUP2/6/8 negatively regulates plant growth and cell size via mediating K^+^ efflux rather than influx (Osakabe et al., 2013; Nieves-Cordones et al., 2016). Another clade IIb gene is AtKUP1/KT1, the first cloned HAK/KT/KUP transporter from *Arabidopsis*; however, its physiological role has not yet been determined (Kim et al., 1998). The role of clade III genes could potentially be discerned based on cotton (*Gossypium hirsutum*) GhKT1, which was specifically upregulated during cotton fiber elongation (Ruan et al., 2001; Nieves-Cordones et al., 2016). As for clade IV transporters, only one have been characterized so far in plants: LjKUP was highly expressed during late nodulation and complemented K^+^-uptake-deficient bacteria (Desbrosses et al., 2004). Finally, PpHAK1 from *Physcomitrella patens* provides insight into clade V function; it regulates steady K^+^ content and plant morphology under non-K^+^-limiting conditions, as well as contributing to high-affinity Rb^+^ and Cs^+^ uptake during K^+^ starvation (Garciadeblas et al., 2007; Nieves-Cordones et al., 2016). In future studies, In future studies, these genes can be functionally characterized for polar localization of auxin in root apex, K^+^ efflux, fiber development, nodule development and plant morphology from IIa, IIc, III, IV and V group members respectively (Figure 1A).

The gene structure and expansion patterns of soybean HAK/KT/KUP genes were highly similar with respect to conserved exon length (commonly 260, 52, and 254 nt), close to the lengths found in poplar (261, 53, and 254 nt) (Figure 1B; He et al., 2012). Segmental duplication was also dominant in both poplar and soybean. Moreover, three WGD events occurred in soybean HAK/KT/KUP evolution; the results were highly similar in poplar (He et al., 2012; Table S2). Ortholog clustering and syntenic analysis of the HAK/KT/KUP family in all five dicots revealed that their last common ancestor had two HAK transporters (Figures S5, S6), confirming the previous work (Gomez-Porras et al., 2012).

In-depth transcriptome profiling via microarray and RNA-seq established the differential expression of 22 HAK/KT/KUP genes for nodulation (Figures 6A,B, and Figure S7). In this family, Glyma19g45260.1 (HAK5), Glyma19g01400.1 (KUP8), Glyma08g39840.1 (KUP12), and Glyma15g17080.3 were maximally expressed for root hair nodulation (Figure 6). Our data corroborates a previous proposal of Glyma19g452601 (HAK5) being a strong candidate for soybean nodulation (De Carvalho et al., 2013), as well as other data describing the role of various transporters in nodulation as described by Collier and Tegeder (2012), Clarke et al. (2014) and Kryvoruchko et al. (2016). However, Glyma08g09720.1 from group IV was not highly expressed for nodulation in soybean, although it was highly similar to the LjKUP gene (in *L. japonicus*) responsible for late nodule development (Desbrosses et al., 2004).

### Voltage-gated K^+^ channels in soybean

The 16 genes encoding voltage-gated K^+^ channels in soybean were higher in number than the known genes in *Arabidopsis* (9), and poplar (11) (Gomez-Porras et al., 2012). The results of our phylogenetic analysis supported previous research (Gomez-Porras et al., 2012; Figure 2A). The maximum number of six K_in_ (AKT1) and three K_in_ (KAT1) putative genes in soybean confirmed the large K^+^ uptake range in soybean, allowing the plant to adjust K^+^ concentrations, especially when exposed to low K^+^ environments (Figure 2A). The recently characterized OsAKT1 from rice confirmed the previously reported, critical role of K_in_ type genes in K^+^ uptake, nutrition, and drought tolerance (Li et al., 2014; Ahmad et al., 2016). Hence, the genes in these clades could be a target for functional characterization, linking them to K^+^ uptake and drought tolerance in soybean and other legumes. Currently, AtKC1 is accepted to be involved in modulating AKT1 expression under low K^+^ stress responses in *Arabidopsis* (Wang et al., 2016). Therefore, we proposed that Glyma06g07840.1 and Glyma04g07750.1 (K_silent_) in soybean have a synergistic effect on AKT1 regulation under K^+^ deficient conditions.

The soybean genome harbors three K_out_ genes in the voltage-gated K^+^ channel family. Glyma05g33660.3 (GORK type) likely influences K^+^ efflux in stomatal closure, as its closest homolog in *Arabidopsis* has already been characterized for stomatal regulation (Hosy et al., 2003). The remaining two genes Glyma14g39330.1 and Glyma02g41040.1 (SKOR type) can be projected for their contribution to shoot-ward K^+^ secretion into the xylem sap (Gaymard et al., 1998). Glyma08g20030.1 and Glyma12g29190.1 (AKT2/3) were identified for weak K^+^ rectification. Functional characterization of the genes from this group has revealed enough functional plasticity to perform different roles in phloem tissue of source and sink organs. Moreover, they are also involved in drought tolerance and salt-induced depolarization of *Arabidopsis* roots (Lacombe et al., 2000; Salvador-Recatala, 2016).

The voltage-gated K^+^ channel family in soybean was observed to have highly conserved gene structure, with lengths of 57, 98, and 185 nt exons across all 16 genes (Figure 2B). Hence, this family probably evolved through these conserved exons. Segmental duplication was dominant during the expansion of this family (Table S2). Only two genes were tandemly duplicated. We also discovered that AKTC1, KAT1, AKT2/3, and GORK/SKOR genes evolved from AKT1 genes around 7–10 mya, a recent gene duplication event in soybean. AKT1 genes were duplicated 9–59 mya, whereas others were duplicated around 188–255 mya, only becoming part of soybean genome later (Table S2). Four rounds of WGD were involved in expanding this family in soybean. Ortholog clustering and syntenic analysis with other five dicots revealed that this family formed one cluster in both the common bean and soybean, while forming two clusters in the other species (Figures S5, S6).

Undoubtedly, voltage-gated K^+^ channels genes were widely expressed in shoots and roots. A detailed microarray and RNA-seq data confirmed their involvement in nodulation (Figure S8 and Figure 6C). Based on our analysis, we proposed that Glyma5g08230.1 (AKT1), Glyma14g39330.1, and Glyma02g41040.1 (SKOR type) are involved in nodule development (Figure 6C). Recently, a candidate gene from *Medicago truncatula* belonging to this family was proposed to affect cell membrane repolarization during the early electrical response to node factor, as well as being involved in stomatal movements and K^+^ secretion into the xylem sap (Damiani et al., 2016). Our microarray and RNA-seq results supported the link to nodule development.

### TPK/KCO family in soybean

Functional TPK channels form dimers comprising two identical subunits; each subunit is characterized by a structure with four TMDs and tandem-pore-forming loops between the first, second, third, and fourth membrane-spanning domains (Maitrejean et al., 2011; Gomez-Porras et al., 2012). Soybean TPK/KCO channels are divided into KCO1, KCO2, and KCO3; these results are similar to Marcel et al. (2010) and Gomez-Porras et al. (2012) (Figure 2A). Findings on KCO1 genes in *Arabidopsis* confirmed that genes in this clade are related to vacuolar K^+^ conductance, salt stress adaptation, and K^+^ homeostasis (Latz et al., 2007; Maitrejean et al., 2011). KCO2 and KCO3 genes form homomeric ion channels *in vivo*; thus, the TPK/KCO genes can be functionally characterized as having such functions (Voelker et al., 2006; Rocchetti et al., 2012). Hence we proposed the putative functions of KCO1, KCO2, and KCO3 genes for vacuolar K^+^ conductance, salt stress adaption and K^+^ homeostasis respectively.

KCO1 genes in soybean did not exhibit any conserved exons and segmentally duplicated around 7 mya (Table S2). One conserved, 147-nt exon was observed in KCO2 and KCO3 genes, suggesting a common ancestor (Marcel et al., 2010; Figure 2B). Moreover, a recent duplication event of 11–14 mya was also found in soybean KCO2 and KCO3 genes (Marcel et al., 2010) (Table S2). Ortholog clustering and syntenic analysis among all five dicot TPK/KCO members revealed only one cluster, similar to results from Marcel et al. (2010) (Figures S5, S6).

Differential expression of Glyma02g46930.1 (KCO1) and Glyma19g40890.1 (KCO3) for nodulation was confirmed through microarray and RNA-seq (Figure S9 and Figure 6D). Until this study, KCO/TPK family had not been annotated for nodulation in any plant.

### HKT family in soybean

The plant HKT family comprises transporters that mediate Na^+^ uptake in roots or in other plant organs. Phylogenetic analysis of HKT genes in soybean grouped them into HKT subfamily 1, highly similar to previous findings (Platten et al., 2006; Gomez-Porras et al., 2012; Figure 3). Recently, GmHKT1;4 in soybean was found to regulate the Na^+^/K^+^ ratio in roots under salt stress (Chen et al., 2014). Therefore, we propose that the three related genes in soybean are also functionally involved in salt stress response. Segmental duplication occurred around 16–28 mya in soybean HKT subfamily 1 (Table S2). Ortholog clustering and syntenic analysis with genes from five dicots showed that they had one common ancestor (one cluster) (Figures S5, S6). Interestingly, we did not find any HKT gene in soybean that was involved in nodulation. Hence, we concluded that this family only affects Na^+^/K^+^ regulation in soybean.

### KEA family in soybean

The novel KEA family is responsible for active K^+^ accumulation and balance, as described by Aranda-Sicilia et al. (2012). Phylogenetic analysis of 12 KEA genes grouped them into three major clades, supporting Chanroj et al. (2012) (Figure 4). KEA1 and KEA2 represent very close homologs, most likely due to a gene-duplication event; thus, they are expected to perform the same transport function (Hohner et al., 2016). Genes KEA4 to KEA6 were all grouped into one clade, again similar to previous work (Chanroj et al., 2012). KEA family genes have been implicated in abiotic stresses, such as K^+^ deficiency, drought, and high salinity (Sheng et al., 2014). Recent studies with KEA1 and KEA2 mutants in *Arabidopsis* underscored their diverse influence in leaf growth and efficient photosynthesis rate (Kunz et al., 2014; Dana et al., 2016). Recently, an electro-neutral KEA3 gene from this family was identified in the thylakoid membrane, predominantly in the *Arabidopsis* stroma lamellae (Armbruster et al., 2014; Kunz et al., 2014). Hence, KEA genes in soybean likely have similar putative functions as their homologs in *Arabidopsis*.

Exon structure among all soybean KEA genes was highly conserved at lengths of 68 and 89 nt, indicating evolution from a common ancestor (Figure 4B). In particular, soybean KEA1 and KEA2 genes were segmentally duplicated 8–34 mya (Table S2) and exhibited similar patterns in phylogeny and gene structure. KEA3, KEA4, KEA5, and KEA6 evolved with a recent genome duplication around 6–7 mya in soybean. Ortholog clustering and syntenic analysis in all five dicots also confirmed that the KEA family was highly conserved throughout their expansion in dicots (Figures S5, S6).

Based on the differential expression observed during root hair inoculation with *B. japonicum*, we propose a new role for KEA2 genes in soybean nodulation (Figure 6E). Specifically, Glyma18g46420.1 and Glyma09g39770.1(KEA2) were differentially expressed in root hair cells 48 HAI mock treated (Figure 6E). RNA-seq data was similar with microarray data and confirmed the differential expression.

## Conclusions

In the soybean genome, we identified 29 HAK/KT/KUP genes, 16 voltage-gated K^+^ channel genes, 9 TPK/KCO channel genes, 4 HKT genes, and 12 KEA genes. Based on phylogenetic analyses, expansion patterns, gene structures, splice sites, paralog analysis, and ortholog clustering, we were able to predict detailed functional properties for several genes (Figures 1–4 and Figures S4–S6). In addition, a microarray analysis clarified the genes' mode and place of expression in focusing on five developmental stages and 68 anatomical parts. Until this study, very little was known about the functional role of these genes in soybean nodulation. Hence, for each family, we proposed models of differential expression related to soybean nodulation, based on microarray and RNA-seq data (Figure 6 and Figures S7–S10).

## Author contributions

HR and MN proposed and wrote the manuscript. ZS and SK managed the graphics. ID, SY, and GC refine and approved the manuscript.

### Conflict of interest statement

The authors declare that the research was conducted in the absence of any commercial or financial relationships that could be construed as a potential conflict of interest.

## Abbreviations

AKT: Arabidopsis K^+^ transporter
ATKC: *Arabidopsis thaliana* K^+^ rectifying channel
BLAST: Basic local alignment search tool
CPA: Cation proton antiporters
GORK: Gated outward rectifying K^+^ channel
HAI: Hours after inoculation
HAK: High-affinity K^+^ transporter
HKT: High-affinity K^+^ transporter
KAT: K^+^ channel in *Arabidopsis thaliana*
KCO: K^+^ channel outward
kDa: Kilo Dalton
KEA: K^+^ efflux antiporters
KT: K^+^ transporter
KUP: + uptake permease
Mya: Million years ago
TPM: Transcripts Per Kilobase Million
SKOR: Stelar K^+^ outward rectifier
SPIK: Shaker pollen inward K^+^ channel
TMDs: Transmembrane domains
TPK: Tandem-pore K^+^ channel
WGD: Whole genome duplication.
