# Supplementary material for: In-Depth Genomic and Transcriptomic Analysis of Five K+ Transporter Gene Families in Soybean Confirm Their Differential Expression for Nodulation
Source: Front Plant Sci. 2017 May 23;8:804. doi: 10.3389/fpls.2017.00804 (PMC5440519; doi:10.3389/fpls.2017.00804)
Supplement: Supplementary file 7 [file DataSheet1.pdf]

Supplementary Material

Title: In-depth genomic and transcriptomic dissection of five potassium (K^+) transporter gene families in soybean confirm their differential expression for nodulation

Hafiz Mamoon Rehman[1], Muhammad Amjad Nawaz[1], Zahid Hussain Shah[2], Sadia Khatoon,[3] Seung Hwan Yang[1]*, Gyuhwa Chung[1]

*** Correspondence:**

Seung Hwan Yang

Tel, +82-10-3603-1260; Fax, +82-61-659-7309; Email: ymichigan@chonna.ac.kr

Gyuhwa Chung

Tel, +82-10-3603-1260; Fax, +82-61-659-7309; Email: chung@chonnam.ac.kr

Supplementary Figure 1

HAK/KT/KUP Family

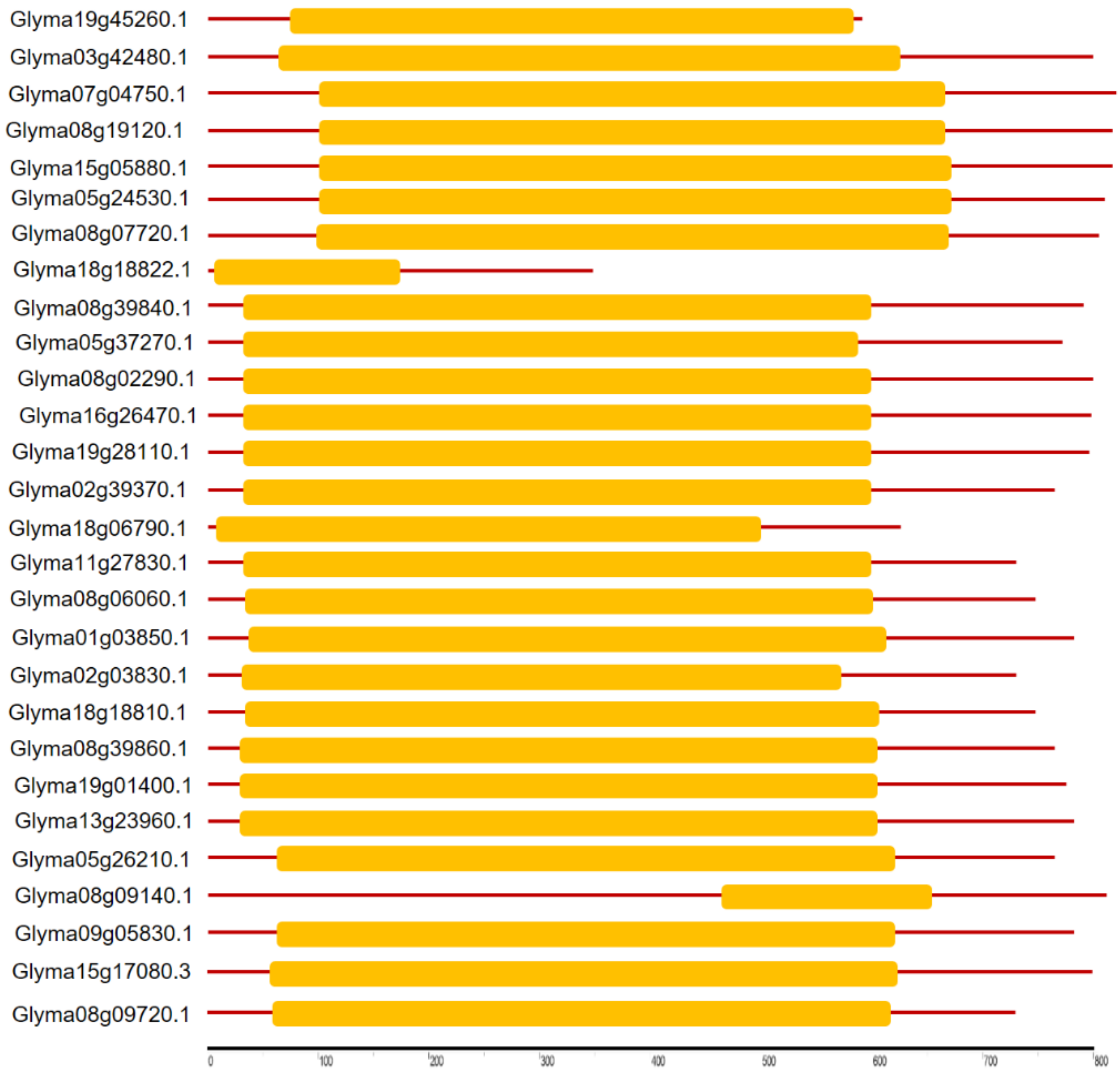

Continue...

Voltage gated K^+ channel family

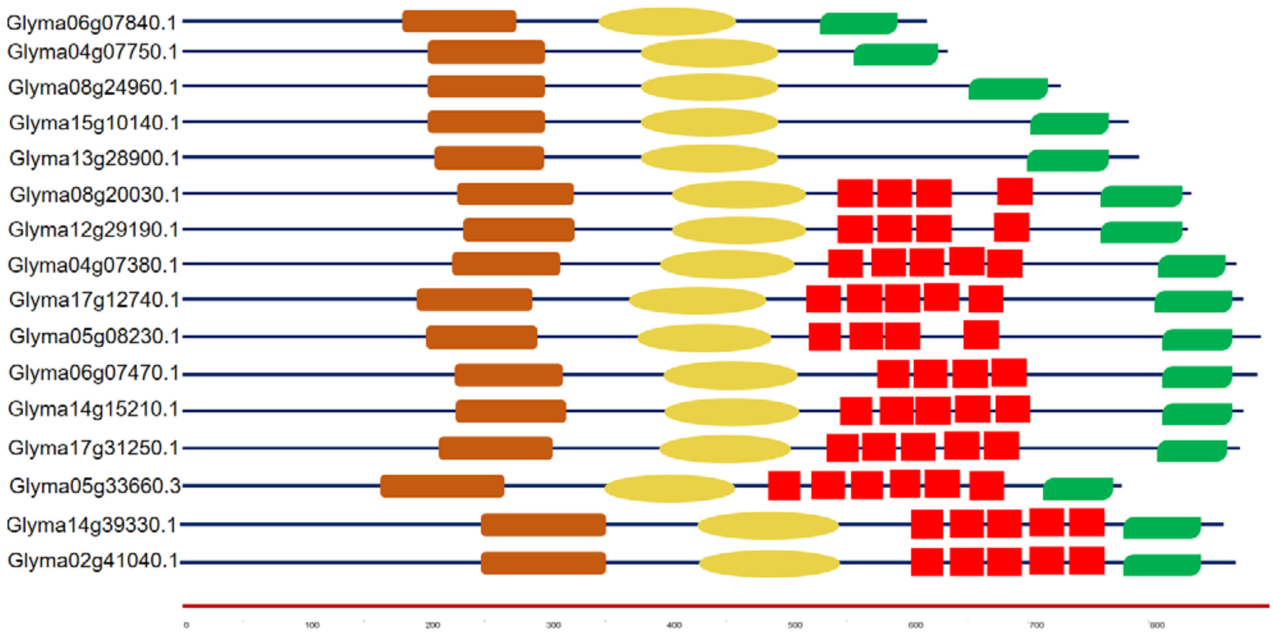

TPK/KCO family

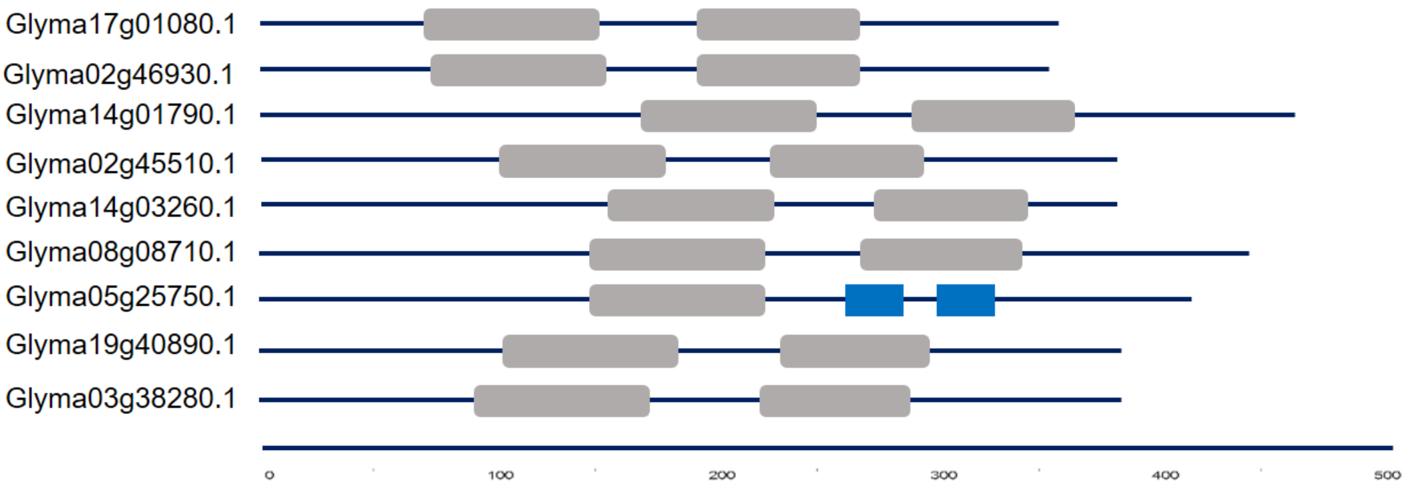

HKT family

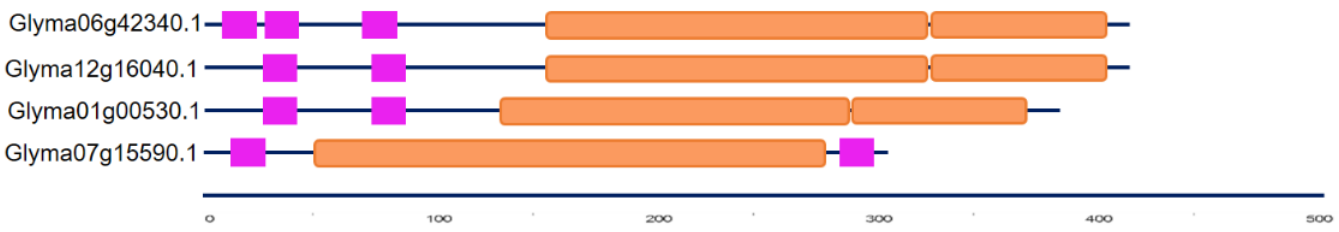

Continue...

KEA family

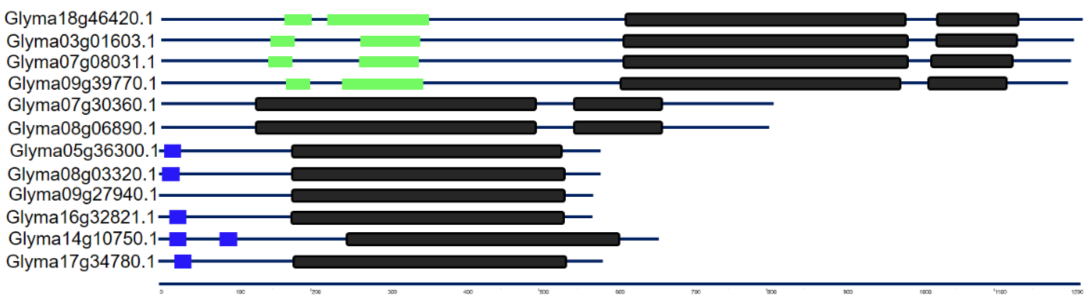

Figure legends

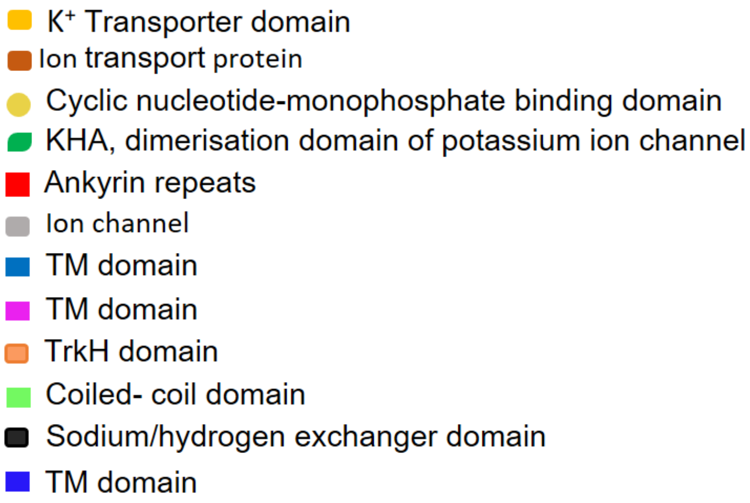

Supplementary Figure 1. Conserved domain organization in all 70 putative K^+ transporter genes from soybean. **HAK/KT/KUP** genes possess a K^+ transporter domain (yellow). The **Voltage-gated channel** family comprises four domains: ion transport protein (dark brown), cyclic nucleotide monophosphate binding domain (light yellow), KHA domain (green), and ankyrin repeats (red). Two ion channels were found in **TPK/KCO** family genes (grey). The **HKT** family has two TrKH and TMD domains (brown

and pink). **KEA** family genes have three coiled-coil domains (light green), a Na^+/H^+ exchanger domain (black), and a TM domain (dark blue).

Supplementary Figure 2

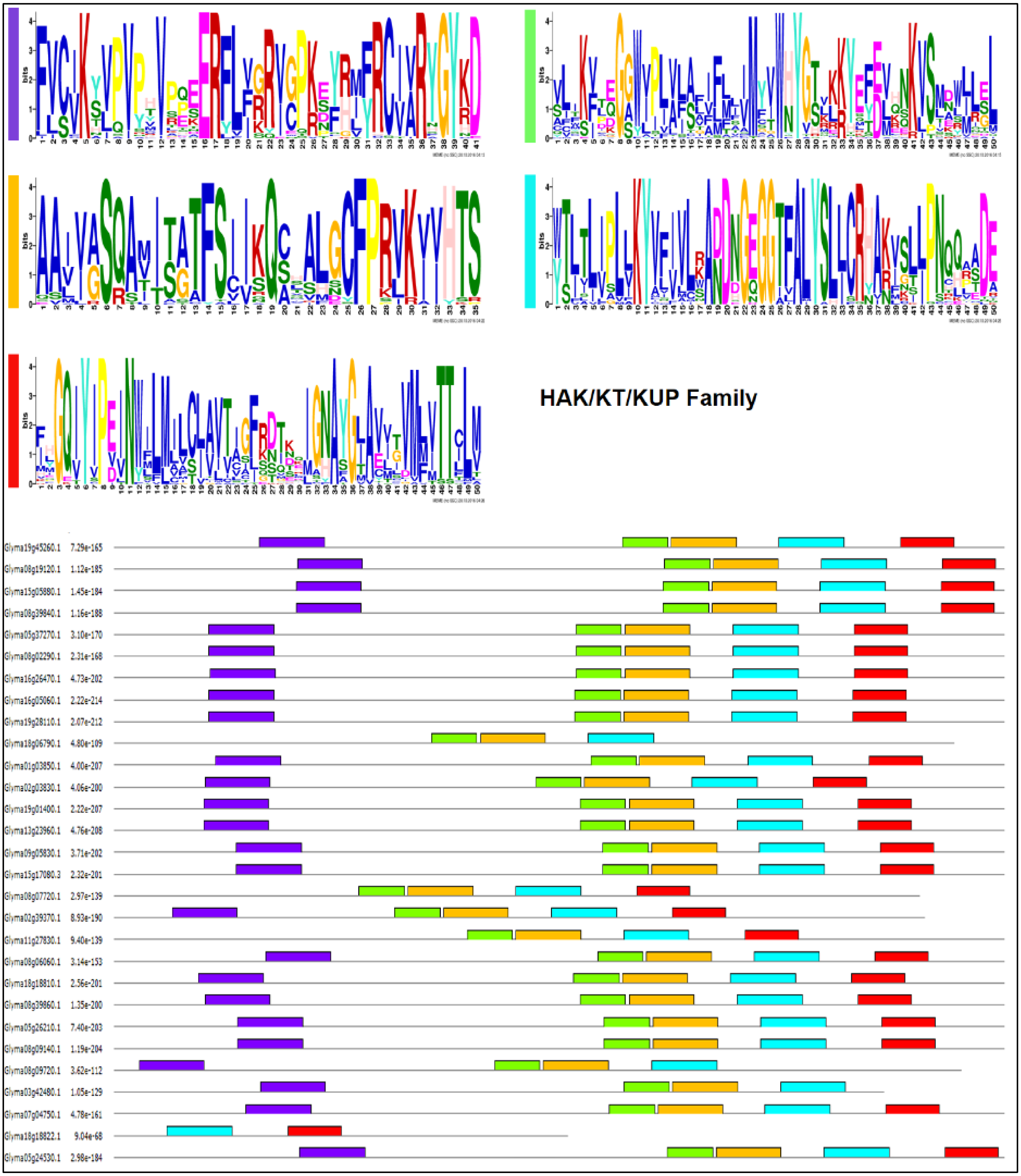

Continue...

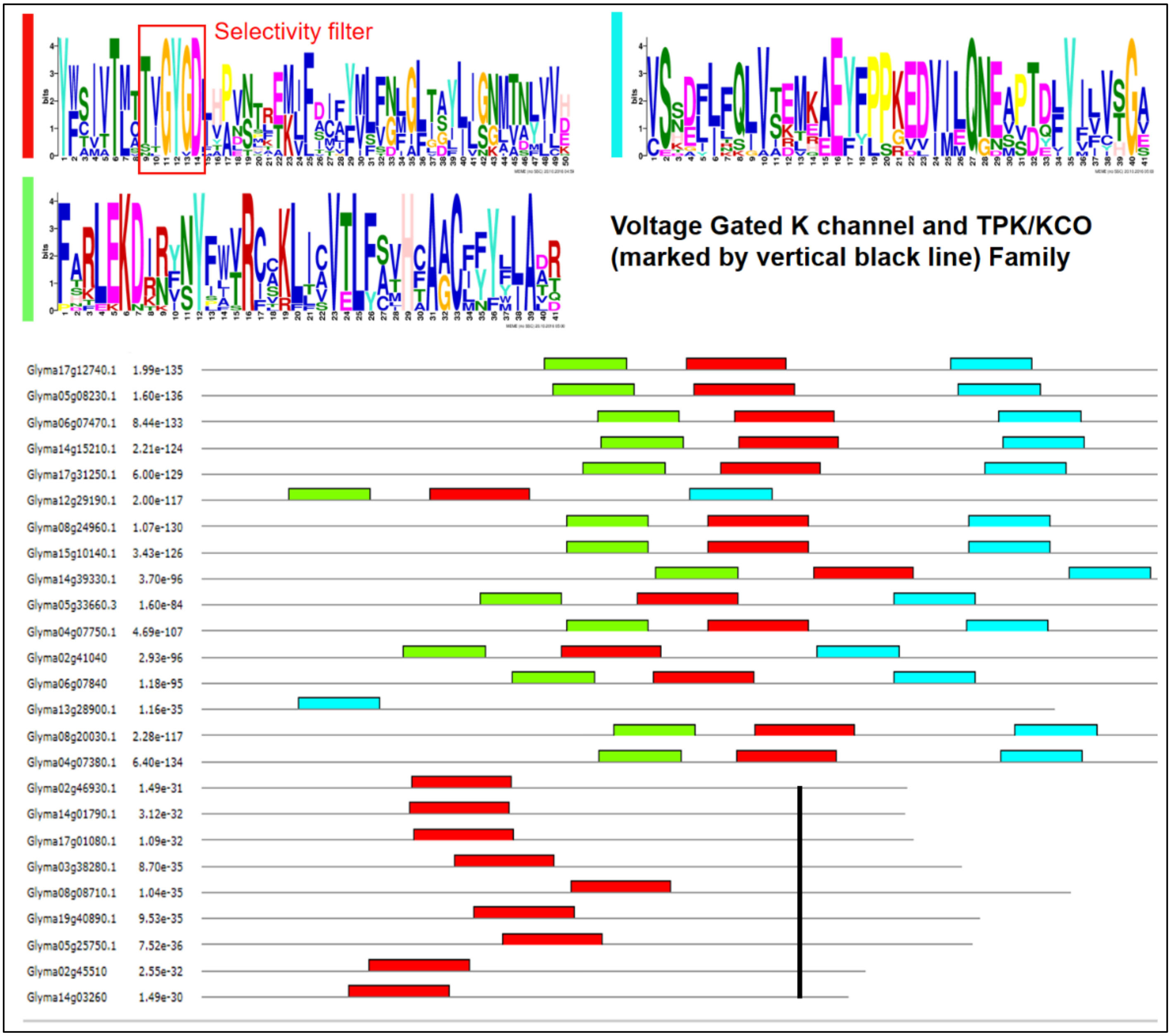

Continue...

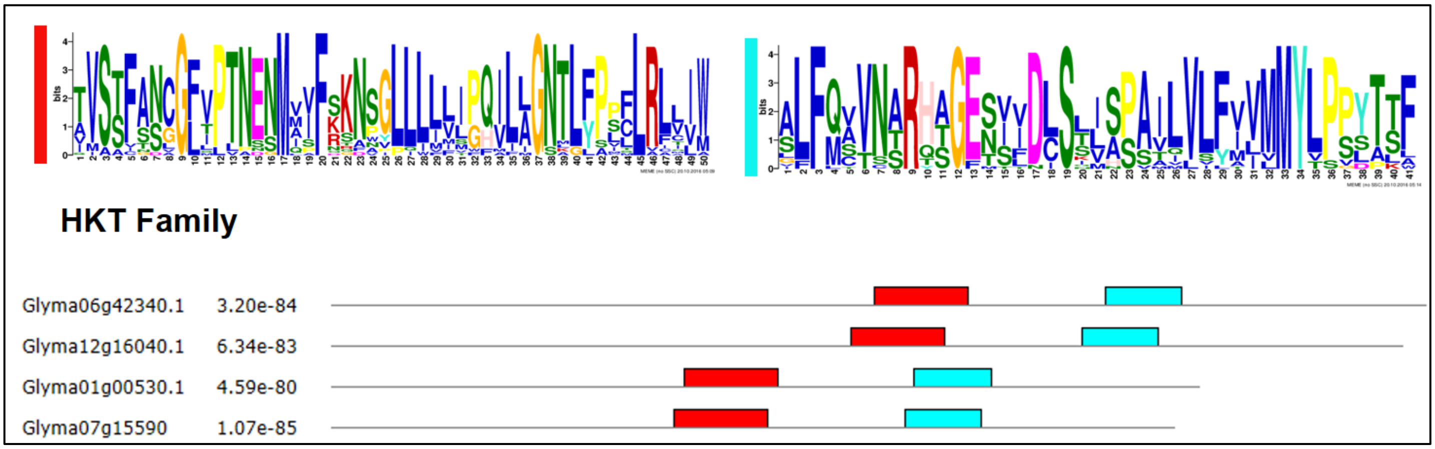

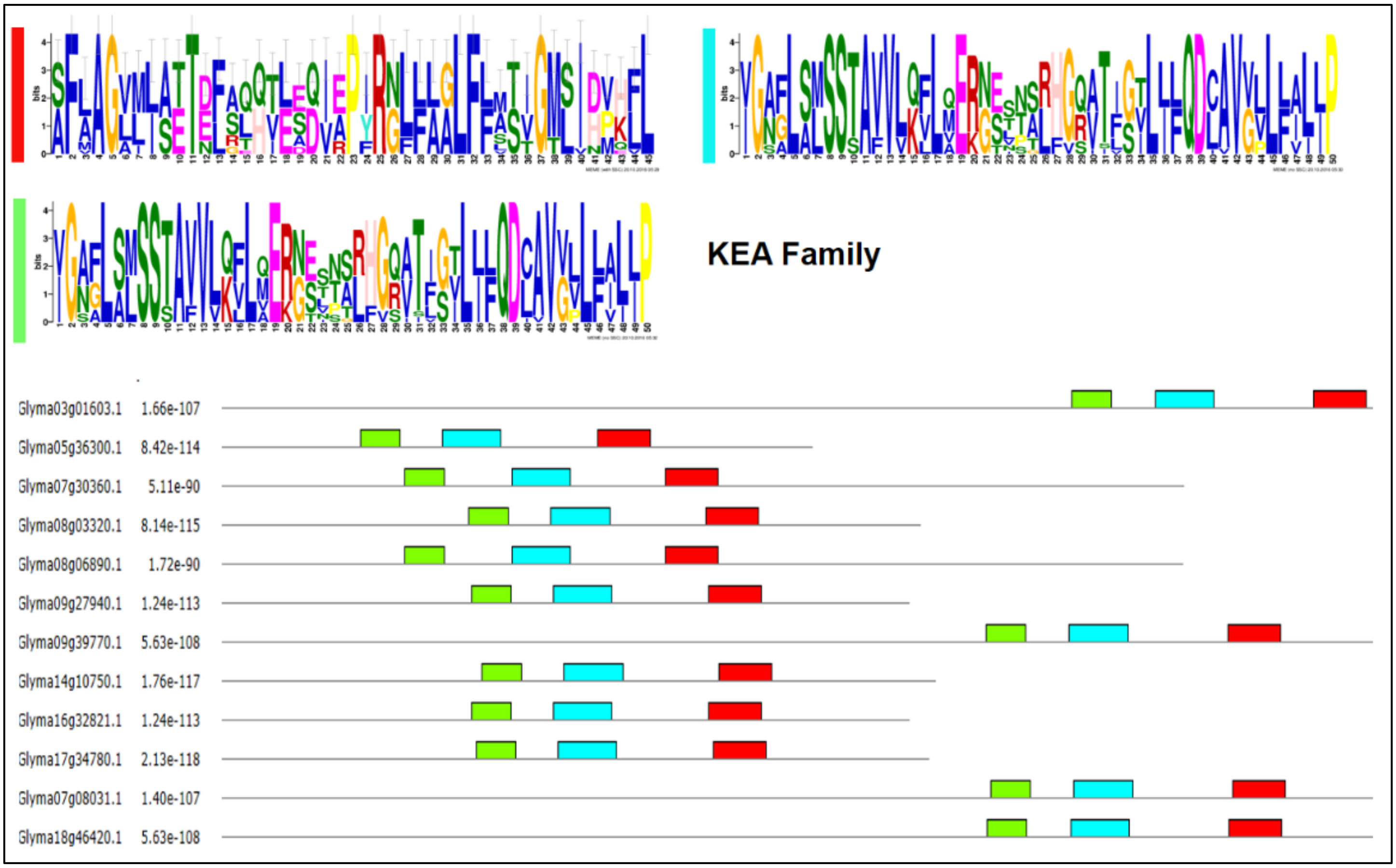

Supplementary Figure 2. Conserved motifs across all 70 putative K^+ transporters genes. **HAK/KT/KUP** family is showing the five highly conserved motifs in their peptide sequences. Voltage-gated and **TPK/KCO** channel family genes have three motifs in which a red color motif having selectivity filter is shown across all the channel proteins. Two and three common motifs are shown for **HKT** and **KEA** family peptides.

Supplementary Figure 3

HAK/KT/KUP family

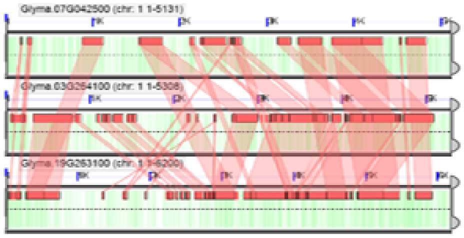

HAK5 duplicated genes

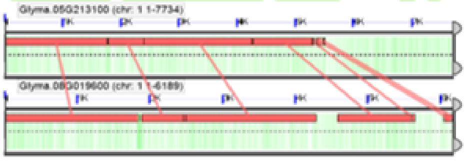

TRH1 duplicated genes

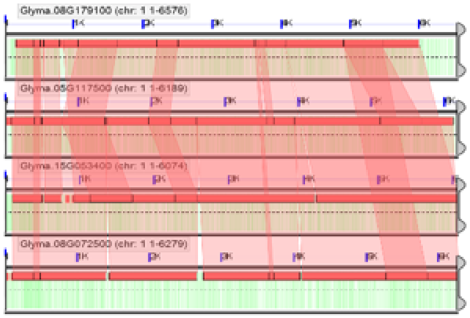

KUP7 duplicated genes

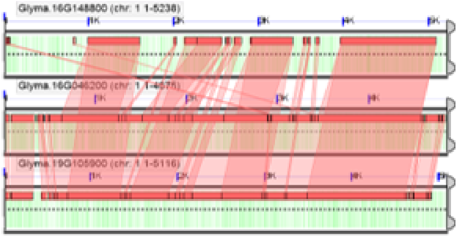

KUP3 duplicated genes

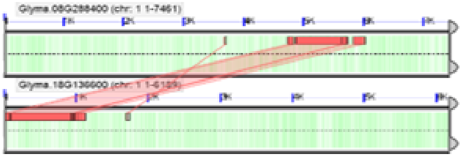

KUP12 duplicated genes

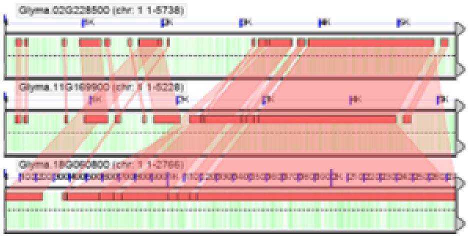

KT1 duplicated genes

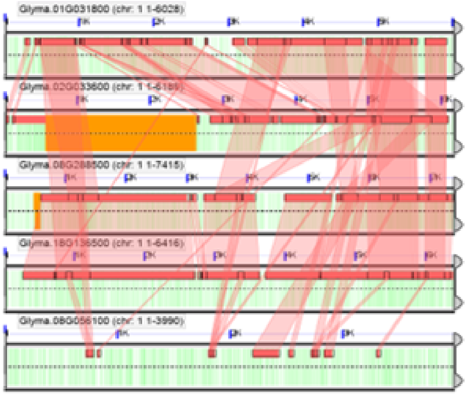

KUP6 duplicated genes

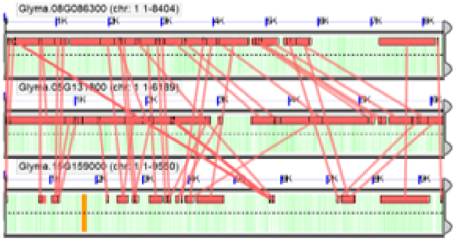

KUP10 duplicated genes

Continue...

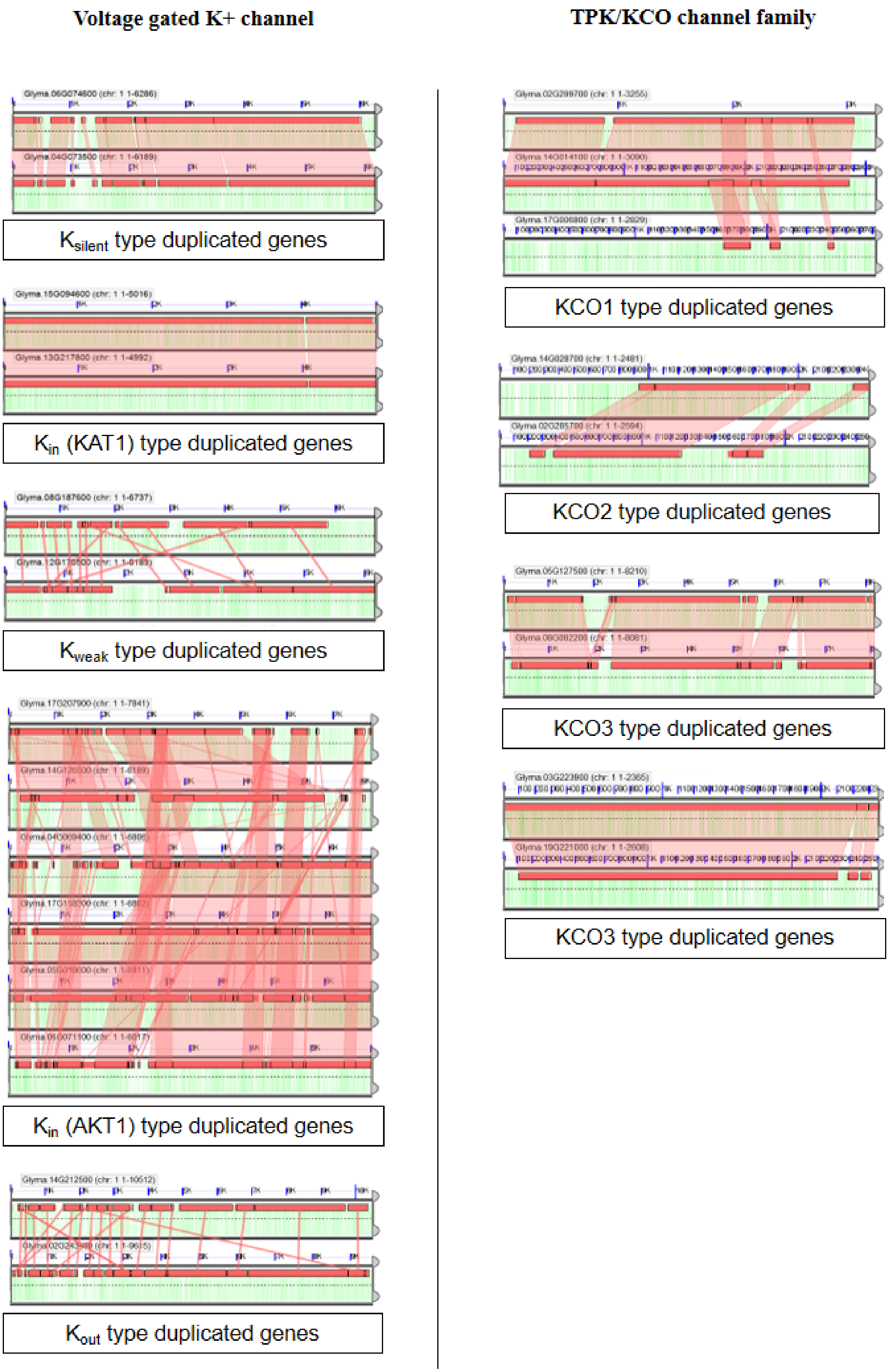
Voltage gated K+ channel
TPK/KCO channel family
K_{silent} type duplicated genes
K_{in} (KAT1) type duplicated genes
K_{weak} type duplicated genes
K_{in} (AKT1) type duplicated genes
K_{out} type duplicated genes
KCO1 type duplicated genes
KCO2 type duplicated genes
KCO3 type duplicated genes
KCO3 type duplicated genes

Continue...

HKT family

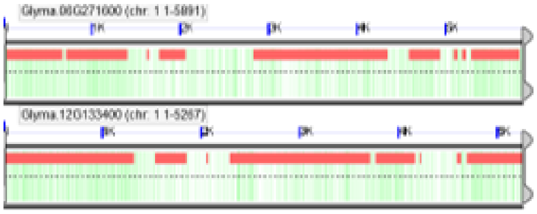

HKT1;1 type duplicated genes

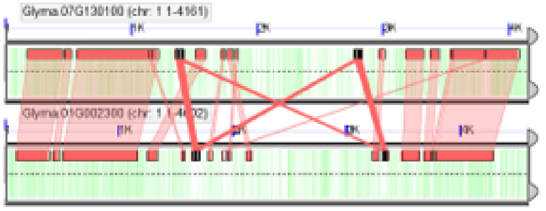

HKT1;3 type duplicated genes

KEA family

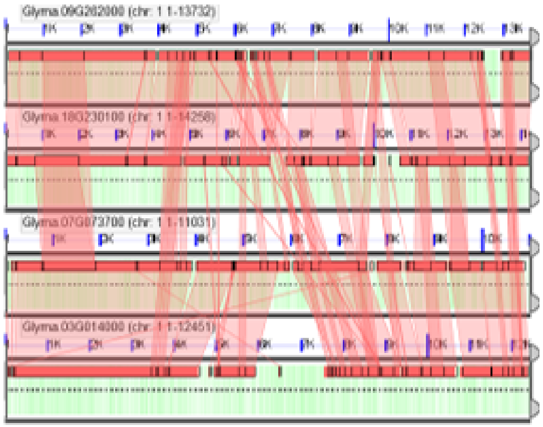

KEA1 and KEA2 duplicated genes

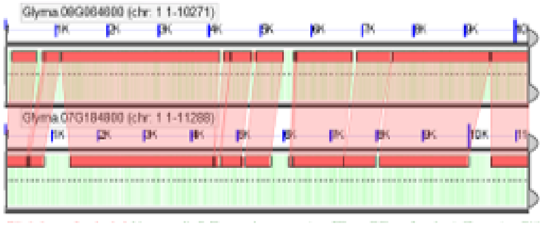

KEA3 duplicated genes

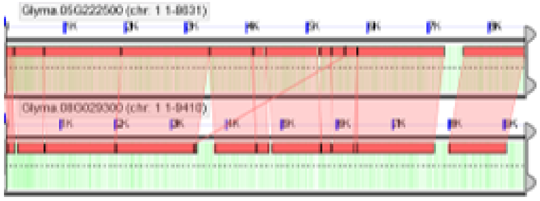

KEA4 duplicated genes

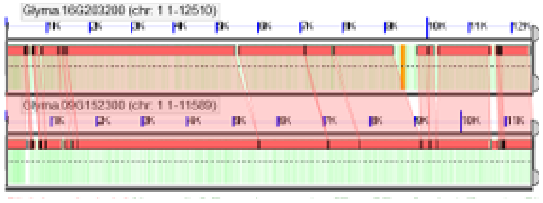

KEA5 duplicated genes

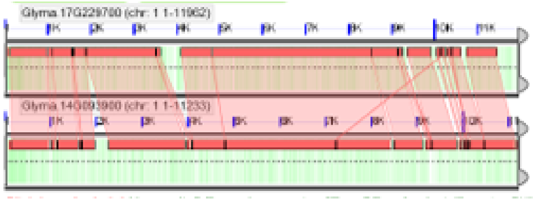

KEA6 duplicated genes

Continue...

Supplementary Figure 3. Paralog and syntenic analysis of all duplicated gene pairs in five K^+ transporter families. Green indicates GC contents in the duplicated gene pairs. Pink indicates the conserved regions during whole genome duplication events in each family.

Supplementary Figure 4

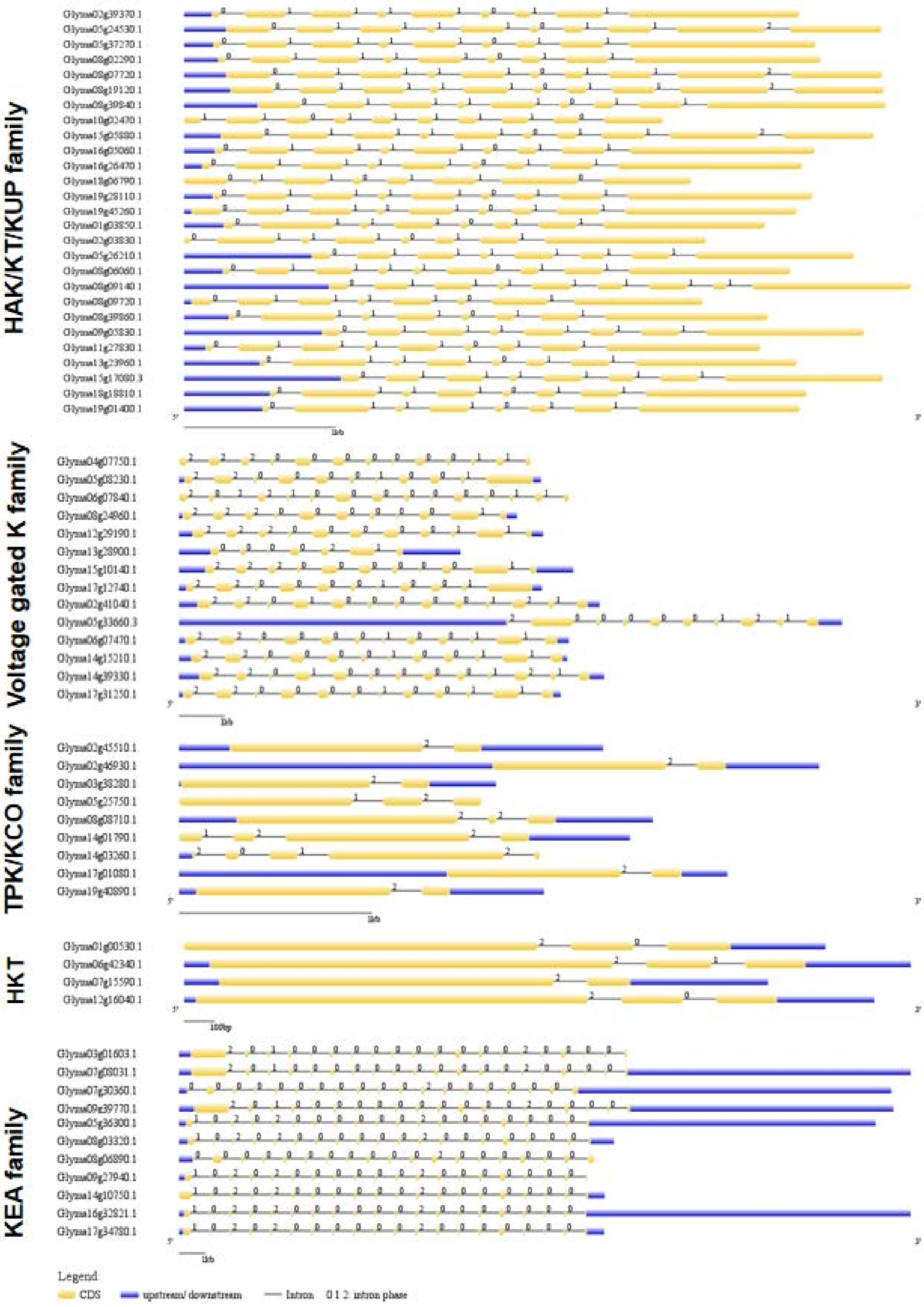

Supplementary Figure 4. Exon-intron structure of all 70 K^+ transporters in soybean. Untranslated regions are shown in blue and introns are shown as grey lines. Yellow indicates the coding sequences of genes. Intron phases are labeled as zero, one, and two.

Supplementary Figure 5

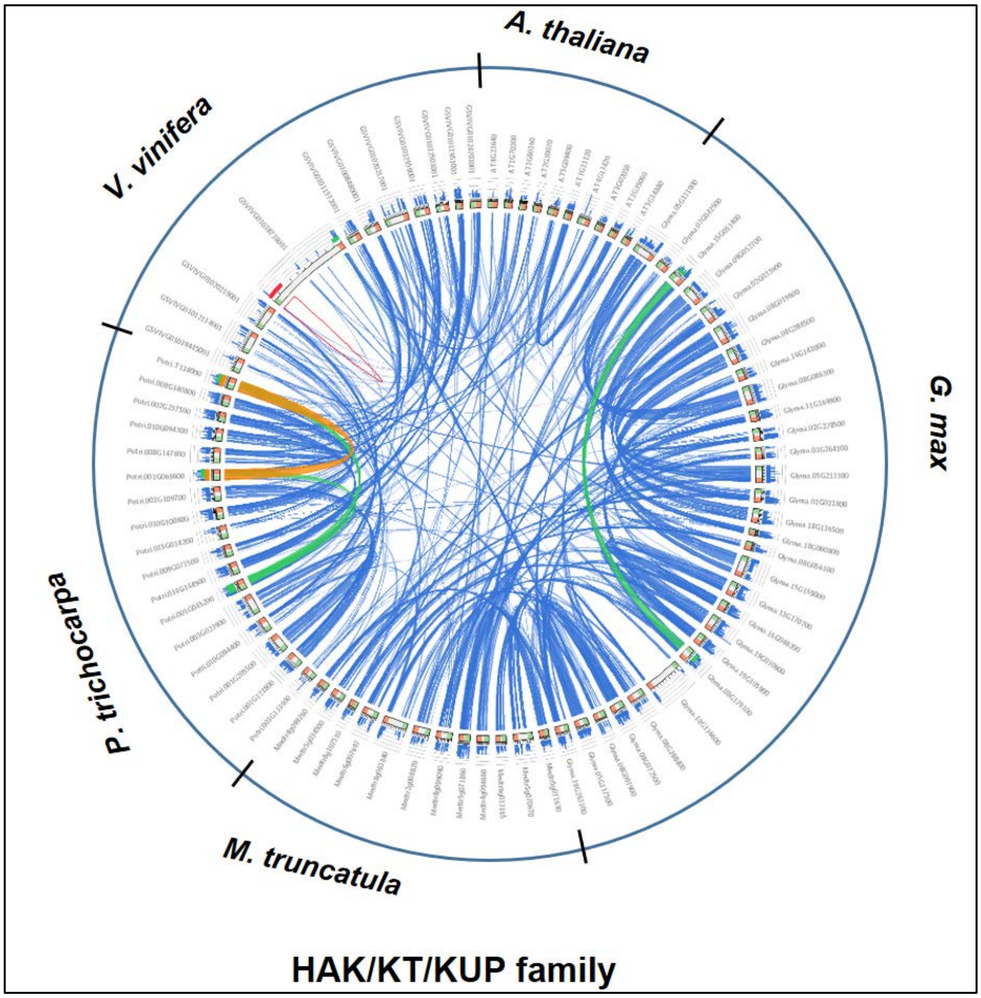

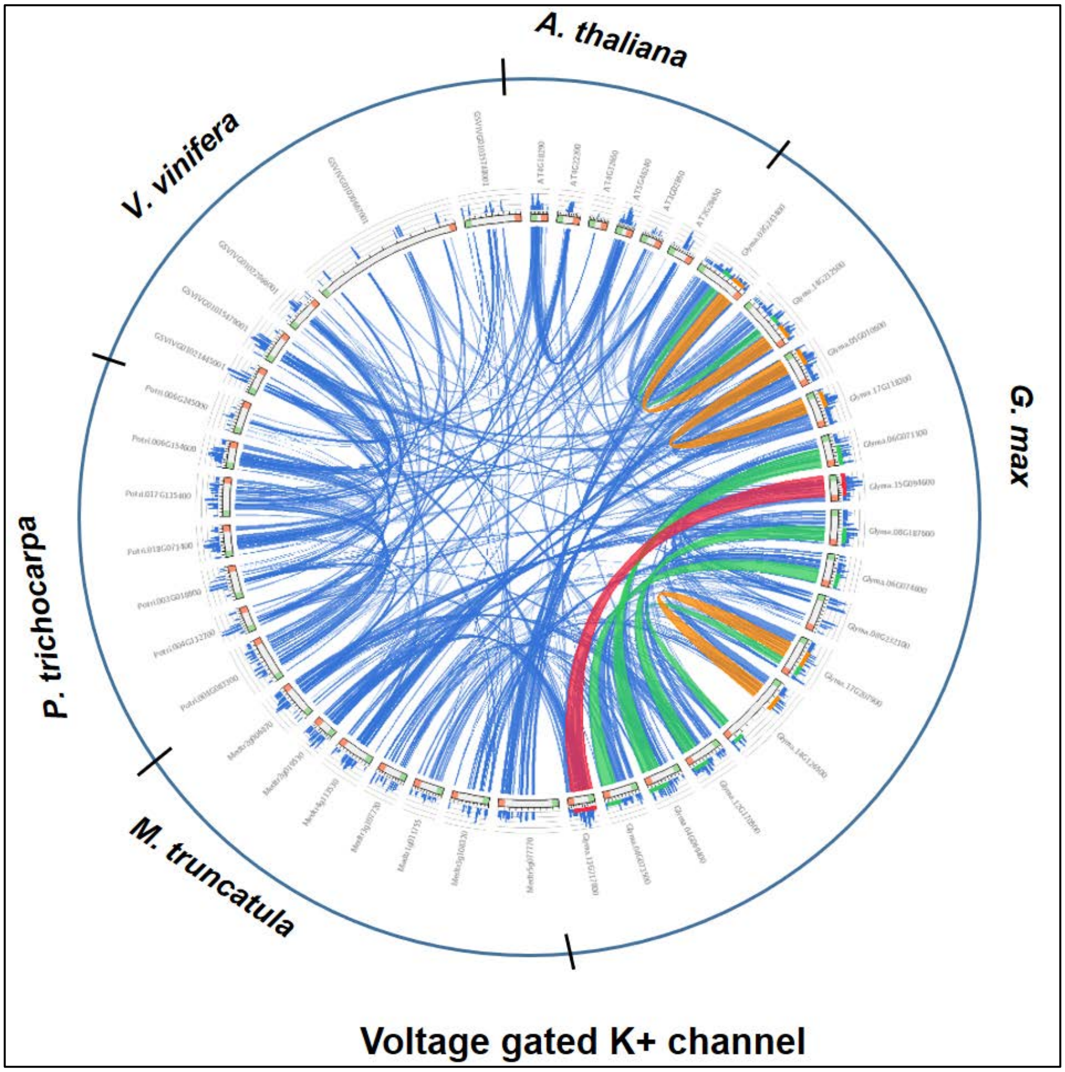
A. thaliana
G. max
M. truncatula
P. trichocarpa
V. vinifera
Voltage gated K+ channel

Continue...

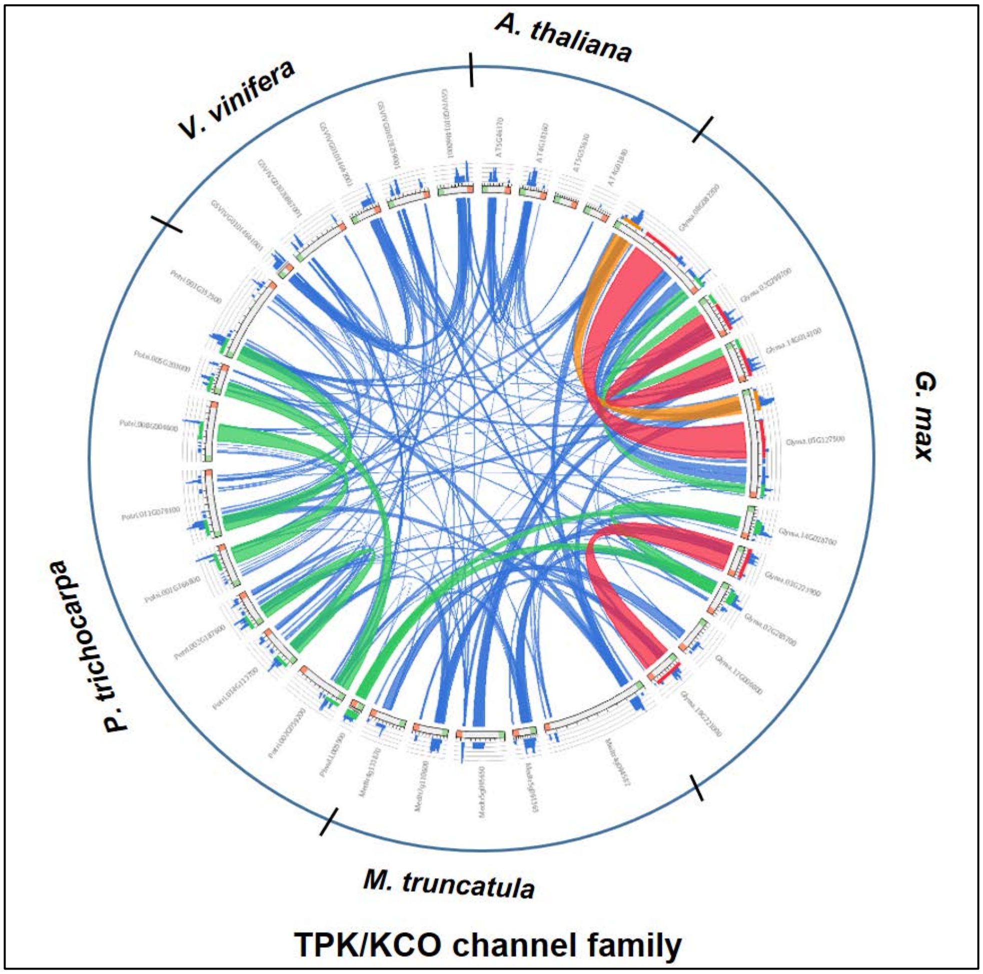
A. thaliana
V. vinifera
G. max
P. trichocarpa
M. truncatula
TPK/KCO channel family

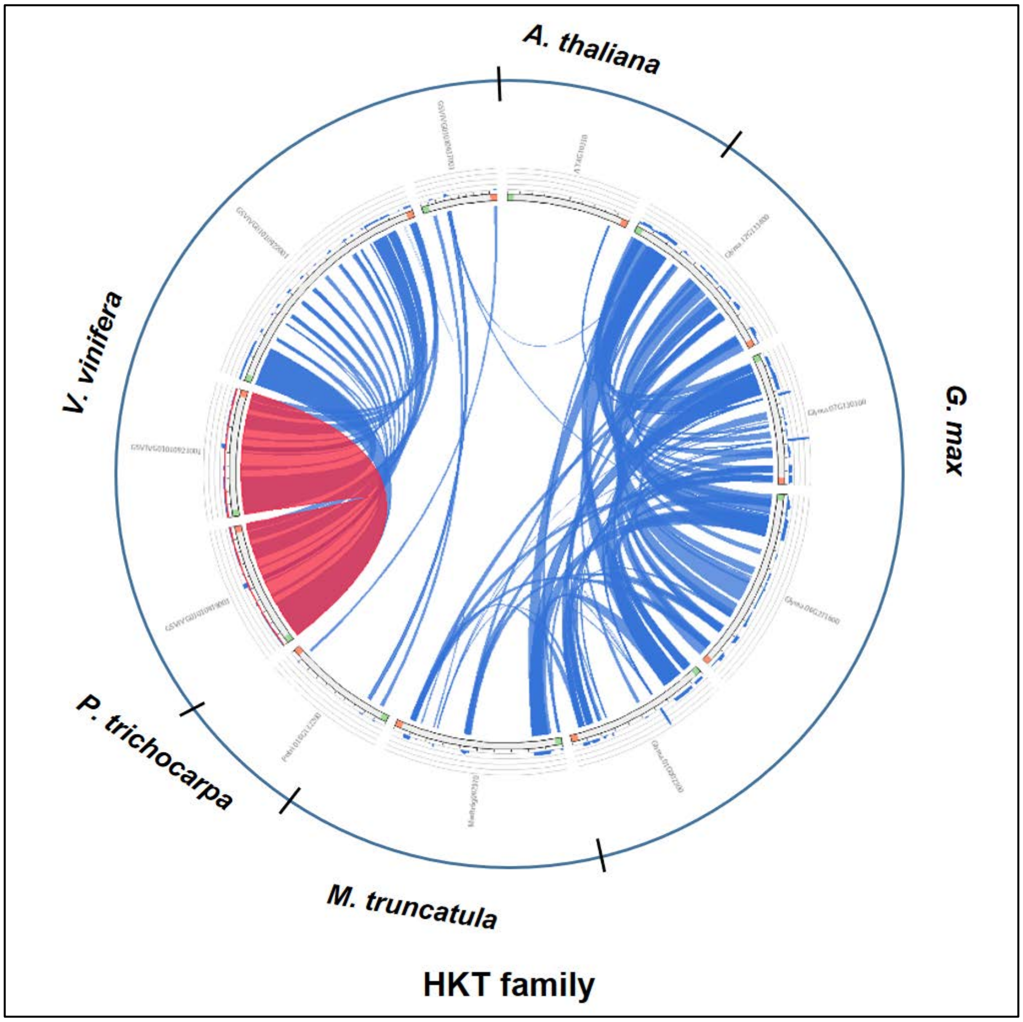
A. thaliana
G. max
M. truncatula
P. trichocarpa
V. vinifera
HKT family

Continue...

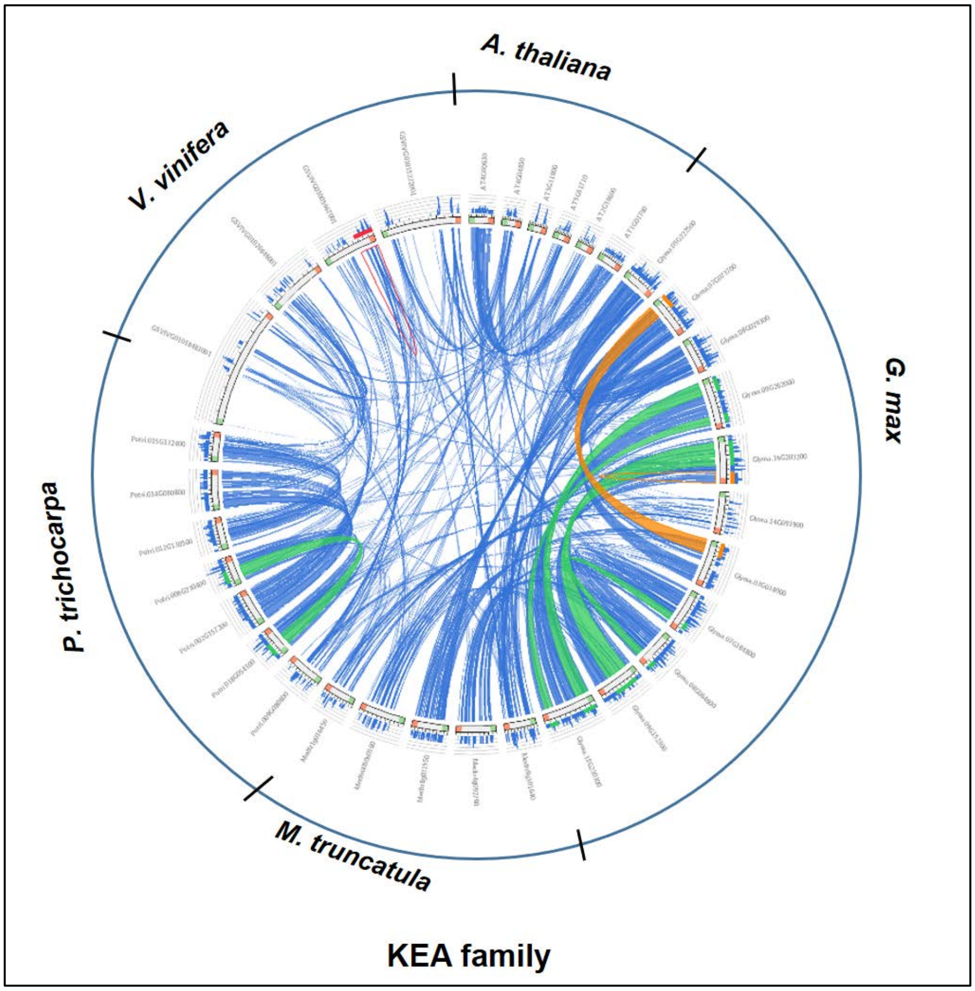

Supplementary Figure 5. Syntenic regions among all the orthologs of five different families identified in five dicots. Almost all annotated protein-coding genes are highly conserved.

Supplementary Figure 6

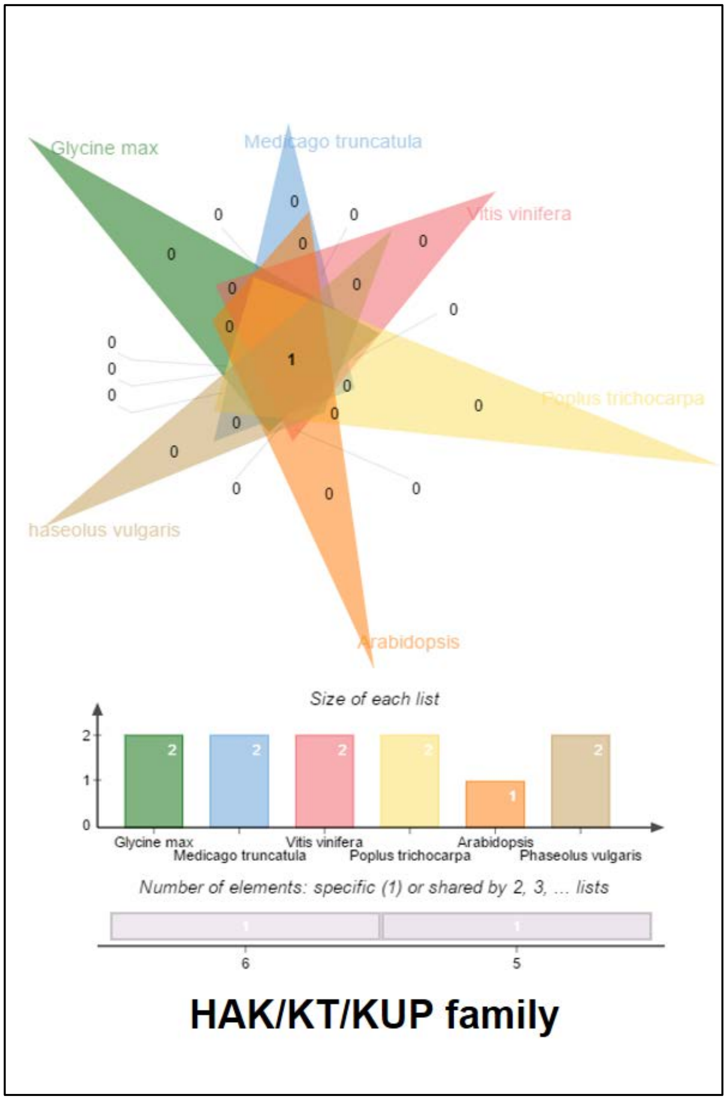

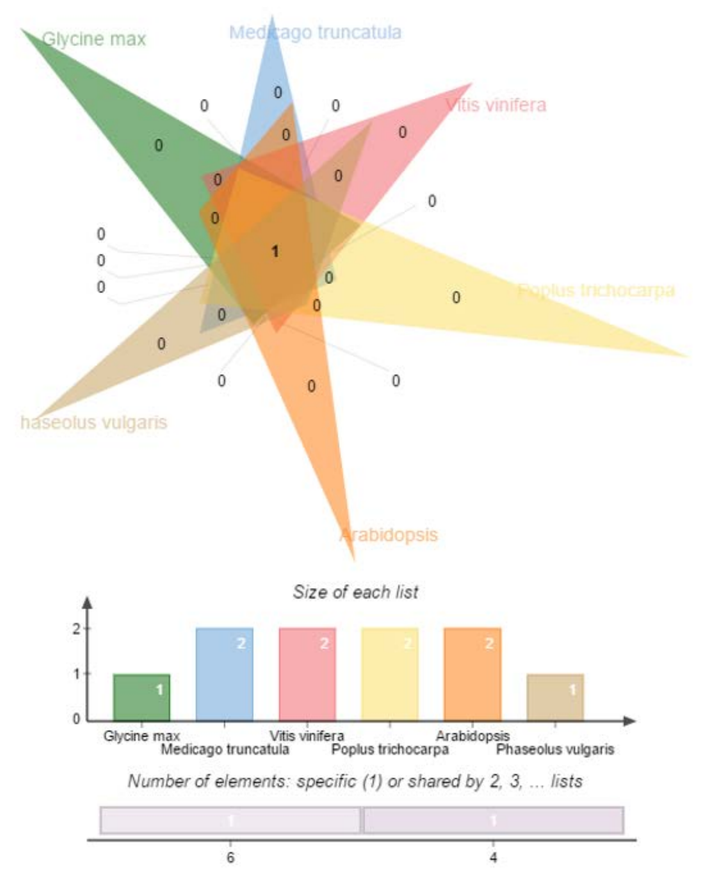

Voltage gated K+ channel

Continue...

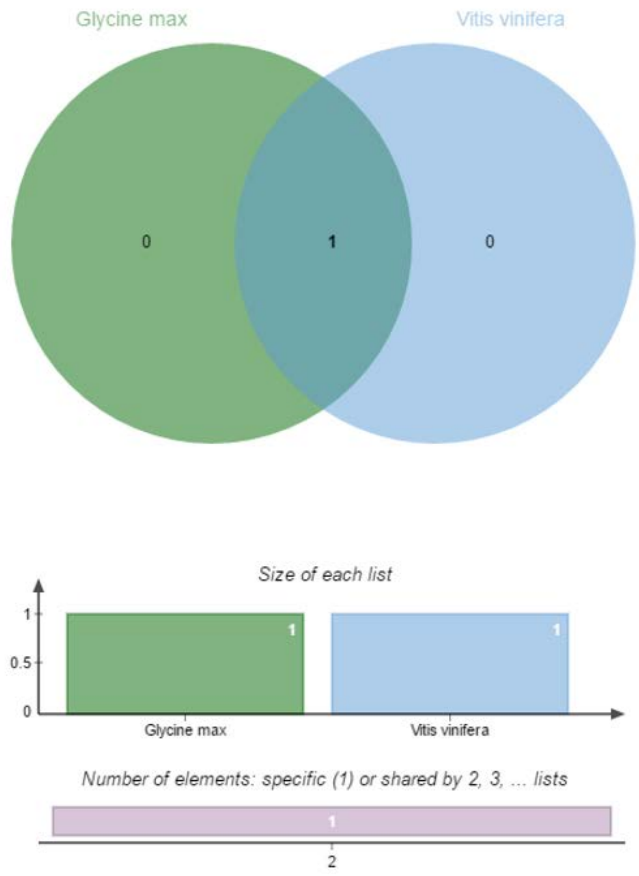
Glycine max
Vitis vinifera
0
1
0
Size of each list
1
0.5
0
1
1
Glycine max
Vitis vinifera
Number of elements: specific (1) or shared by 2, 3, ... lists
1
2

HKT family

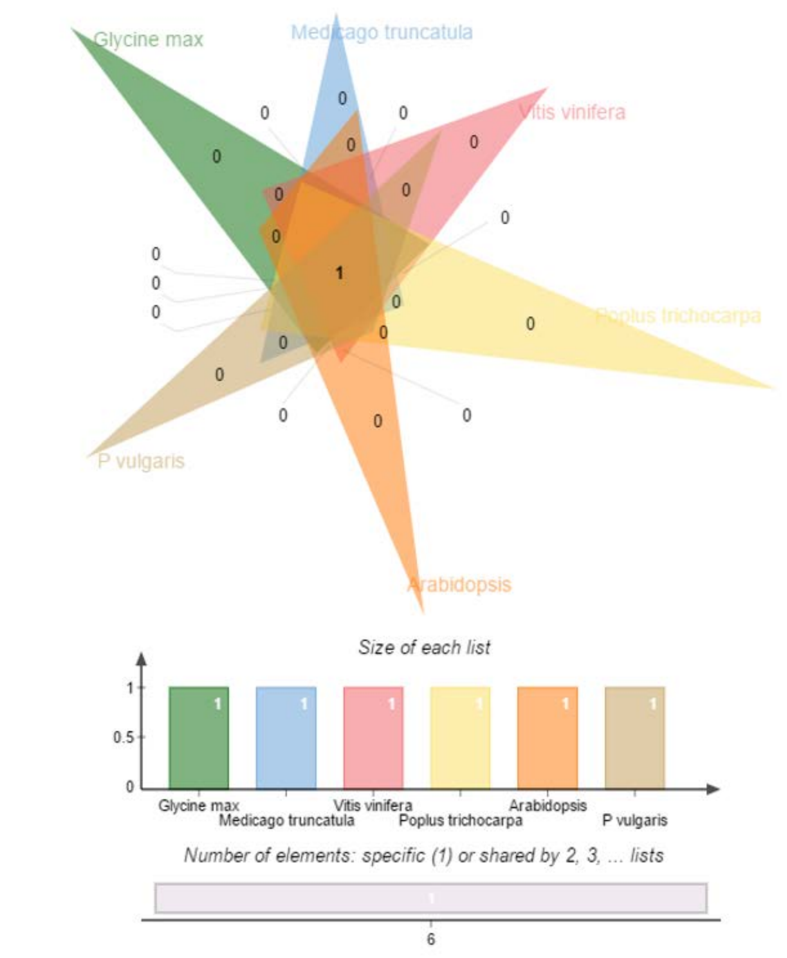

TPK/KCO family

Continue...

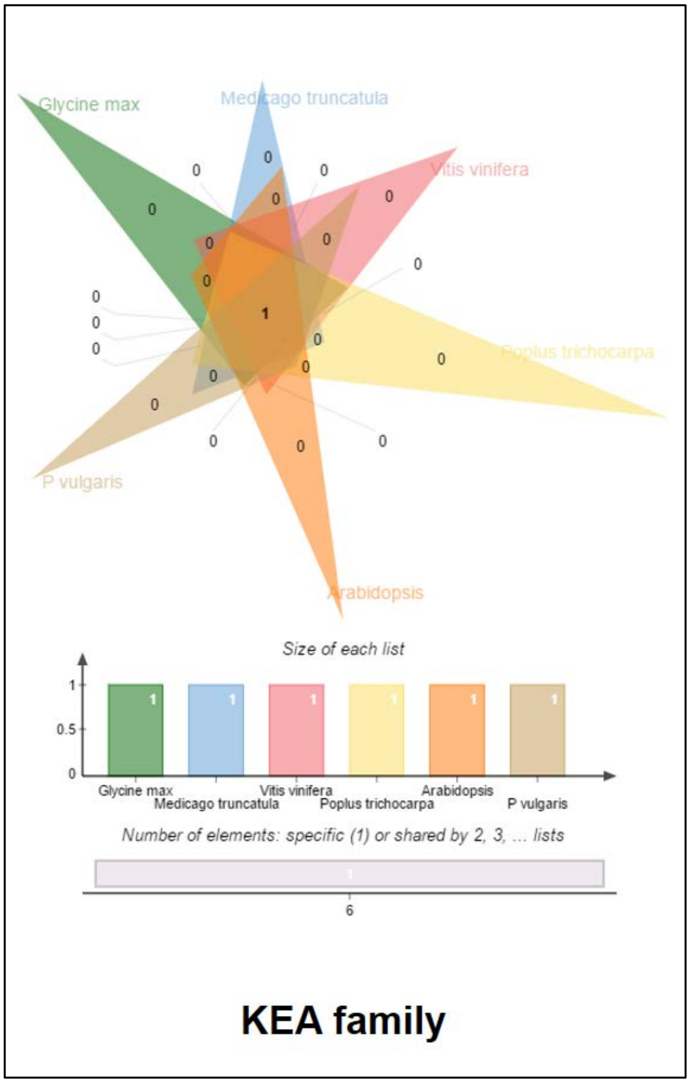

Supplementary Figure 6. Ortholog clustering of five K^+ transporter gene families in five dicots. Number of clusters can be observed in the given figures.

Supplementary Figure 7

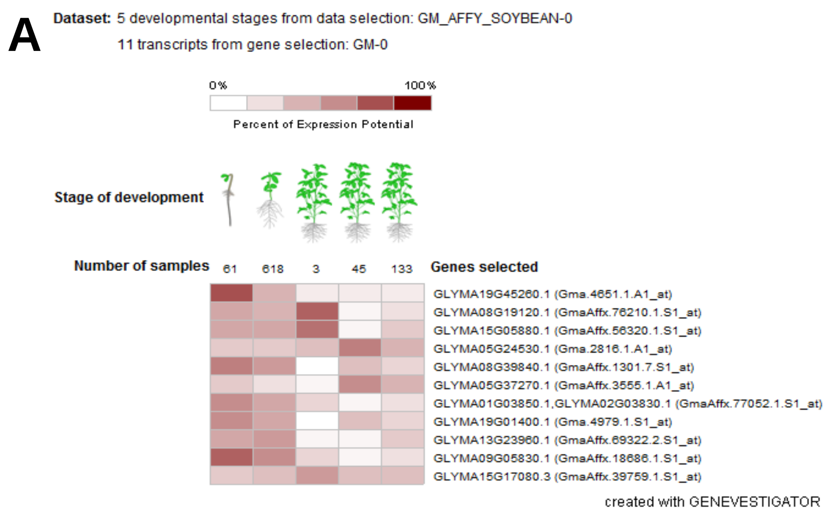

B

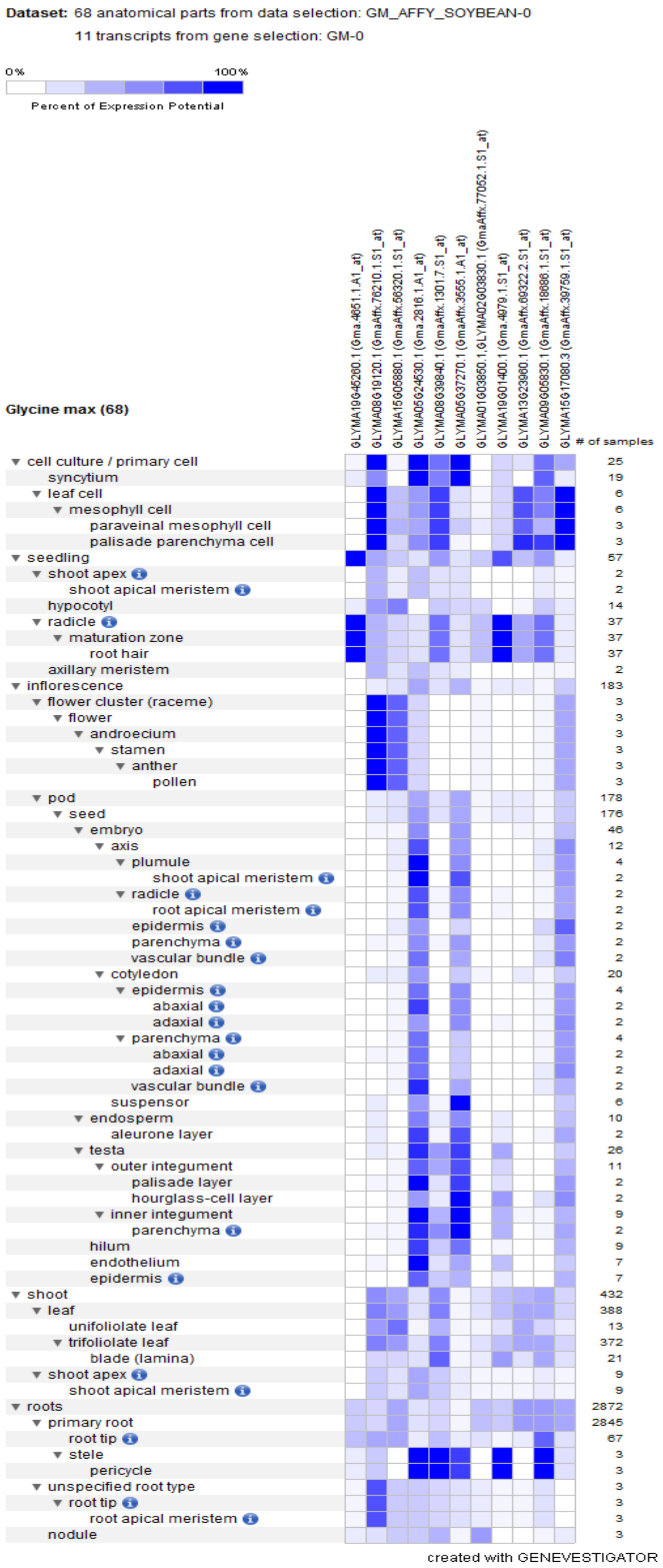
Dataset: 68 anatomical parts from data selection: GM_AFFY_SOYBEAN-0
11 transcripts from gene selection: GM-0
0%
100%
Percent of Expression Potential
Glycine max (68)
GLYMA19G45260.1 (Gma.4651.1.A1_at)
GLYMA08G19120.1 (GmaAffx.76210.1.S1_at)
GLYMA15G05880.1 (GmaAffx.56320.1.S1_at)
GLYMA05G24530.1 (Gma.2816.1.A1_at)
GLYMA08G39840.1 (GmaAffx.1301.7.S1_at)
GLYMA05G37270.1 (GmaAffx.3555.1.A1_at)
GLYMA01G03850.1,GLYMA02G03830.1 (GmaAffx.77052.1.S1_at)
GLYMA19G01400.1 (Gma.4979.1.S1_at)
GLYMA13G23960.1 (GmaAffx.69322.2.S1_at)
GLYMA09G05830.1 (GmaAffx.18686.1.S1_at)
GLYMA15G17080.3 (GmaAffx.39759.1.S1_at)
# of samples
cell culture / primary cell 25
syncytium 19
leaf cell 6
mesophyll cell 6
paraveinal mesophyll cell 3
palisade parenchyma cell 3
seedling 57
shoot apex 2
shoot apical meristem 2
hypocotyl 14
radicle 37
maturation zone 37
root hair 37
axillary meristem 2
inflorescence 183
flower cluster (raceme) 3
flower 3
androecium 3
stamen 3
anther 3
pollen 3
pod 178
seed 176
embryo 46
axis 12
plumule 4
shoot apical meristem 2
radicle 2
root apical meristem 2
epidermis 2
parenchyma 2
vascular bundle 2
cotyledon 20
epidermis 4
abaxial 2
adaxial 2
parenchyma 4
abaxial 2
adaxial 2
vascular bundle 2
suspensor 6
endosperm 10
aleurone layer 2
testa 26
outer integument 11
palisade layer 2
hourglass-cell layer 2
inner integument 9
parenchyma 2
hilum 9
endothelium 7
epidermis 7
shoot 432
leaf 388
unifoliolate leaf 13
trifoliolate leaf 372
blade (lamina) 21
shoot apex 9
shoot apical meristem 9
roots 2872
primary root 2845
root tip 67
stele 3
pericycle 3
unspecified root type 3
root tip 3
root apical meristem 3
nodule 3
created with GENEVESTIGATOR

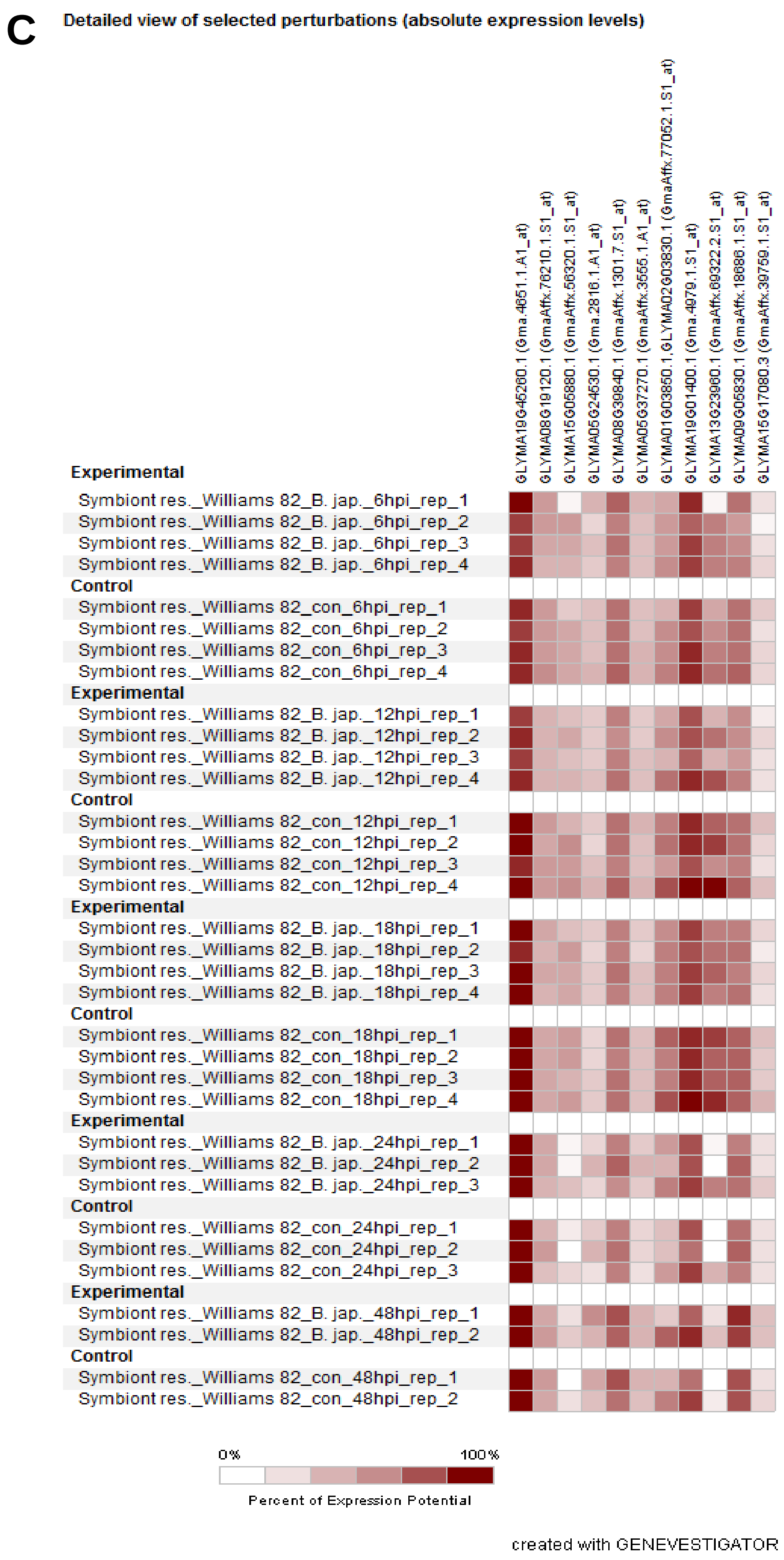
C
Detailed view of selected perturbations (absolute expression levels)
GLYMA19G45260.1 (Gma.4651.1.A1_at)
GLYMA08G19120.1 (GmaAffx.78210.1.S1_at)
GLYMA15G05880.1 (GmaAffx.56320.1.S1_at)
GLYMA05G24530.1 (Gma.2816.1.A1_at)
GLYMA08G39840.1 (GmaAffx.1301.7.S1_at)
GLYMA05G37270.1 (GmaAffx.3555.1.A1_at)
GLYMA01G03850.1,GLYMA02G03830.1 (GmaAffx.77052.1.S1_at)
GLYMA19G01400.1 (Gma.4979.1.S1_at)
GLYMA13G23960.1 (GmaAffx.69322.2.S1_at)
GLYMA09G05830.1 (GmaAffx.18886.1.S1_at)
GLYMA15G17080.3 (GmaAffx.39759.1.S1_at)
Experimental
Symbiont res._Williams 82_B. jap._6hpi_rep_1
Symbiont res._Williams 82_B. jap._6hpi_rep_2
Symbiont res._Williams 82_B. jap._6hpi_rep_3
Symbiont res._Williams 82_B. jap._6hpi_rep_4
Control
Symbiont res._Williams 82_con_6hpi_rep_1
Symbiont res._Williams 82_con_6hpi_rep_2
Symbiont res._Williams 82_con_6hpi_rep_3
Symbiont res._Williams 82_con_6hpi_rep_4
Experimental
Symbiont res._Williams 82_B. jap._12hpi_rep_1
Symbiont res._Williams 82_B. jap._12hpi_rep_2
Symbiont res._Williams 82_B. jap._12hpi_rep_3
Symbiont res._Williams 82_B. jap._12hpi_rep_4
Control
Symbiont res._Williams 82_con_12hpi_rep_1
Symbiont res._Williams 82_con_12hpi_rep_2
Symbiont res._Williams 82_con_12hpi_rep_3
Symbiont res._Williams 82_con_12hpi_rep_4
Experimental
Symbiont res._Williams 82_B. jap._18hpi_rep_1
Symbiont res._Williams 82_B. jap._18hpi_rep_2
Symbiont res._Williams 82_B. jap._18hpi_rep_3
Symbiont res._Williams 82_B. jap._18hpi_rep_4
Control
Symbiont res._Williams 82_con_18hpi_rep_1
Symbiont res._Williams 82_con_18hpi_rep_2
Symbiont res._Williams 82_con_18hpi_rep_3
Symbiont res._Williams 82_con_18hpi_rep_4
Experimental
Symbiont res._Williams 82_B. jap._24hpi_rep_1
Symbiont res._Williams 82_B. jap._24hpi_rep_2
Symbiont res._Williams 82_B. jap._24hpi_rep_3
Control
Symbiont res._Williams 82_con_24hpi_rep_1
Symbiont res._Williams 82_con_24hpi_rep_2
Symbiont res._Williams 82_con_24hpi_rep_3
Experimental
Symbiont res._Williams 82_B. jap._48hpi_rep_1
Symbiont res._Williams 82_B. jap._48hpi_rep_2
Control
Symbiont res._Williams 82_con_48hpi_rep_1
Symbiont res._Williams 82_con_48hpi_rep_2
0%
100%
Percent of Expression Potential
created with GENEVESTIGATOR

Supplementary Figure 7. Microarray analysis of HAK/KT/KUP family genes. **(A)** Genes expression across five developmental stages. **(B)** Genes expression in 68 anatomical parts. **(C)** Detailed view of genes expressed for *B. japnicum* inoculation to root hairs for nodulation in soybean.

Supplementary Figure 8

A

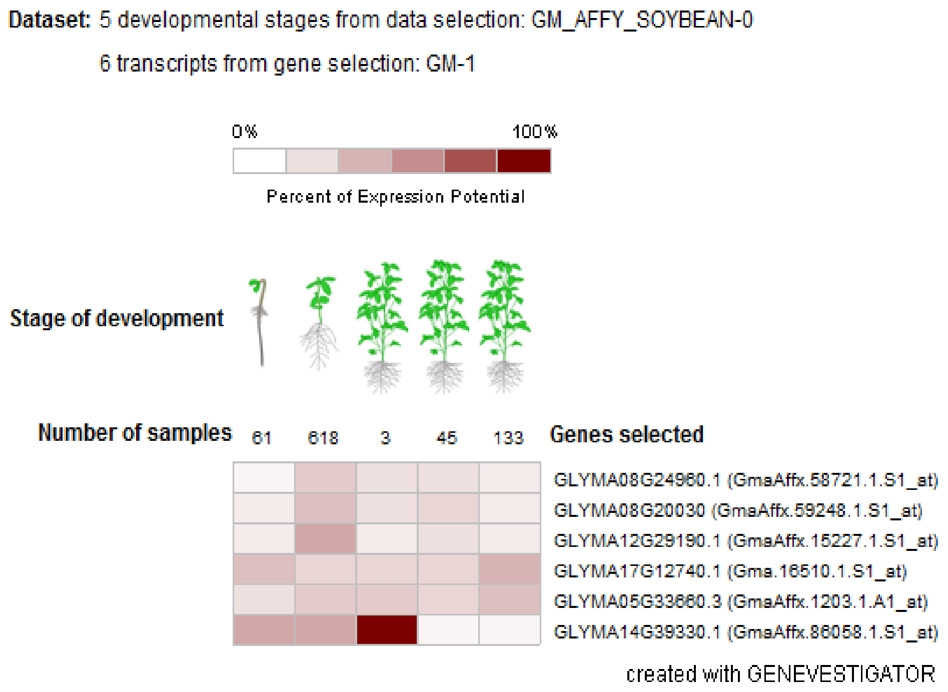

B

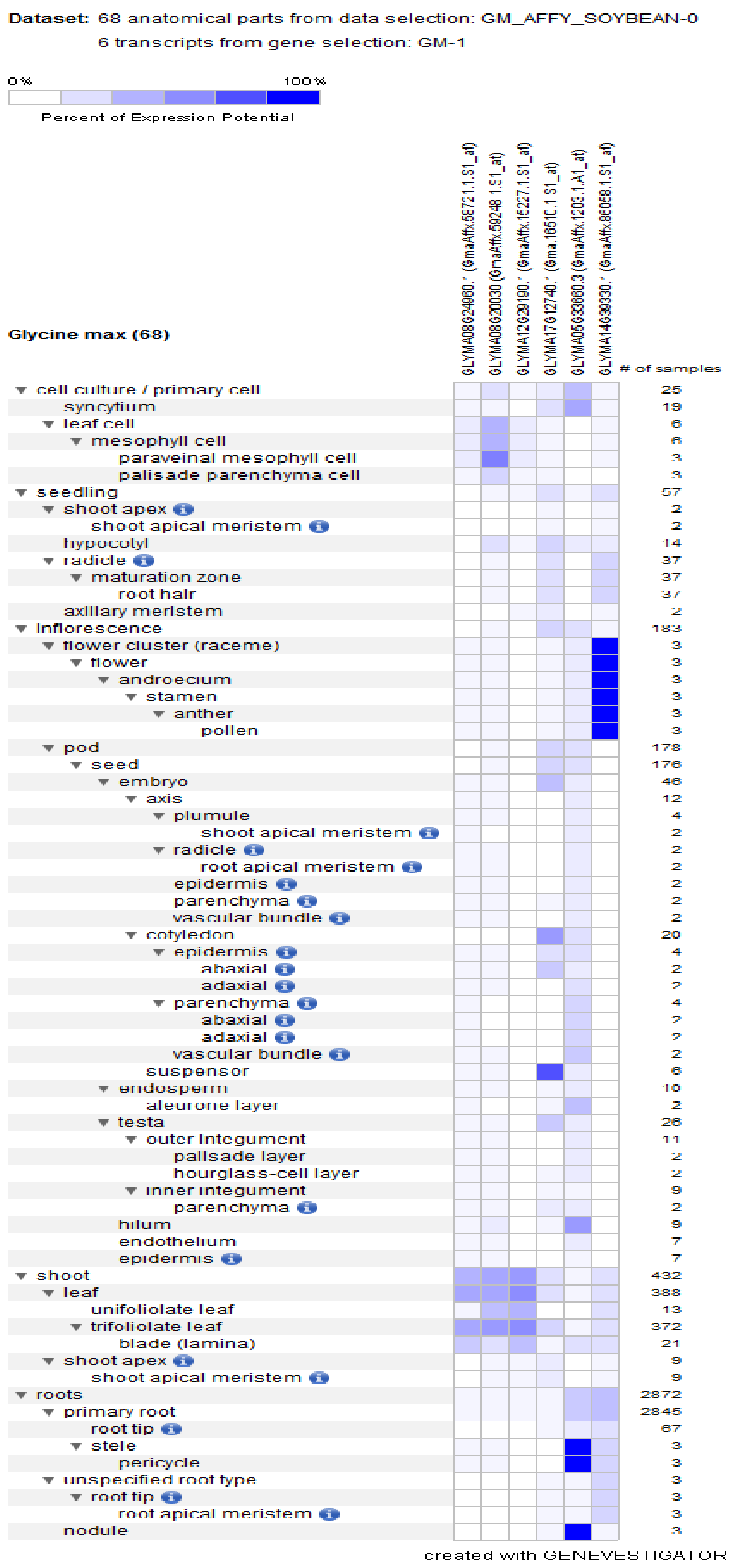
Dataset: 68 anatomical parts from data selection: GM_AFFY_SOYBEAN-0
6 transcripts from gene selection: GM-1
0%
100%
Percent of Expression Potential
Glycine max (68)
GLYMA08G24060.1 (GmaAffx.58721.1.S1_at)
GLYMA08G20030 (GmaAffx.59248.1.S1_at)
GLYMA12G29190.1 (GmaAffx.15227.1.S1_at)
GLYMA17G12740.1 (Gma.16510.1.S1_at)
GLYMA05G33680.3 (GmaAffx.1203.1.A1_at)
GLYMA14G39330.1 (GmaAffx.86058.1.S1_at)
# of samples
cell culture / primary cell 25
syncytium 19
leaf cell 6
mesophyll cell 6
paraveinal mesophyll cell 3
palisade parenchyma cell 3
seedling 57
shoot apex 2
shoot apical meristem 2
hypocotyl 14
radicle 37
maturation zone 37
root hair 37
axillary meristem 2
inflorescence 183
flower cluster (raceme) 3
flower 3
androecium 3
stamen 3
anther 3
pollen 3
pod 178
seed 176
embryo 46
axis 12
plumule 4
shoot apical meristem 2
radicle 2
root apical meristem 2
epidermis 2
parenchyma 2
vascular bundle 2
cotyledon 20
epidermis 4
abaxial 2
adaxial 2
parenchyma 4
abaxial 2
adaxial 2
vascular bundle 2
suspensor 6
endosperm 10
aleurone layer 2
testa 26
outer integument 11
palisade layer 2
hourglass-cell layer 2
inner integument 9
parenchyma 2
hilum 9
endothelium 7
epidermis 7
shoot 432
leaf 388
unifoliolate leaf 13
trifoliolate leaf 372
blade (lamina) 21
shoot apex 9
shoot apical meristem 9
roots 2872
primary root 2846
root tip 67
stele 3
pericycle 3
unspecified root type 3
root tip 3
root apical meristem 3
nodule 3
created with GENEVESTIGATOR

C

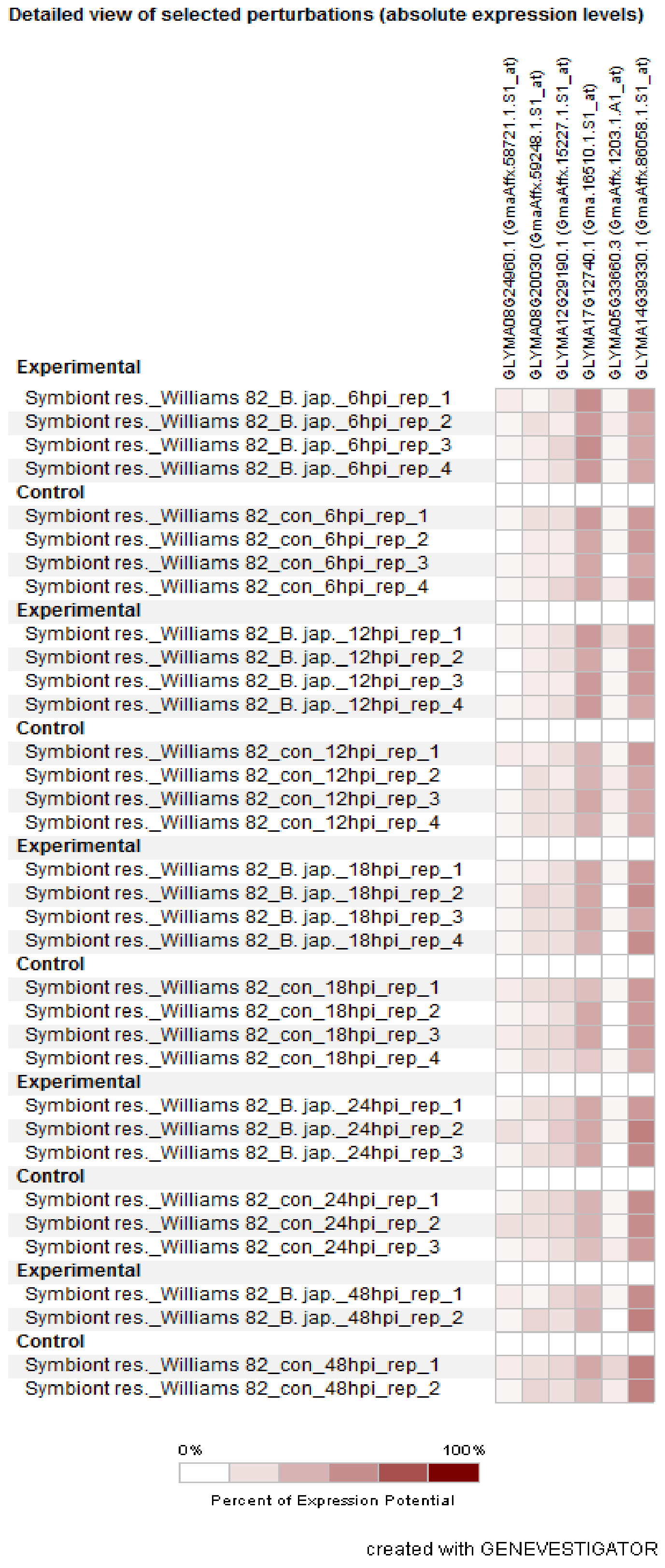
Detailed view of selected perturbations (absolute expression levels)
GLYMA08G24960.1 (GmaAffx.58721.1.S1_at)
GLYMA08G20030 (GmaAffx.59248.1.S1_at)
GLYMA12G29190.1 (GmaAffx.15227.1.S1_at)
GLYMA17G12740.1 (Gma.16510.1.S1_at)
GLYMA05G33660.3 (GmaAffx.1203.1.A1_at)
GLYMA14G39330.1 (GmaAffx.86058.1.S1_at)
Experimental
Symbiont res._Williams 82_B. jap._6hpi_rep_1
Symbiont res._Williams 82_B. jap._6hpi_rep_2
Symbiont res._Williams 82_B. jap._6hpi_rep_3
Symbiont res._Williams 82_B. jap._6hpi_rep_4
Control
Symbiont res._Williams 82_con_6hpi_rep_1
Symbiont res._Williams 82_con_6hpi_rep_2
Symbiont res._Williams 82_con_6hpi_rep_3
Symbiont res._Williams 82_con_6hpi_rep_4
Experimental
Symbiont res._Williams 82_B. jap._12hpi_rep_1
Symbiont res._Williams 82_B. jap._12hpi_rep_2
Symbiont res._Williams 82_B. jap._12hpi_rep_3
Symbiont res._Williams 82_B. jap._12hpi_rep_4
Control
Symbiont res._Williams 82_con_12hpi_rep_1
Symbiont res._Williams 82_con_12hpi_rep_2
Symbiont res._Williams 82_con_12hpi_rep_3
Symbiont res._Williams 82_con_12hpi_rep_4
Experimental
Symbiont res._Williams 82_B. jap._18hpi_rep_1
Symbiont res._Williams 82_B. jap._18hpi_rep_2
Symbiont res._Williams 82_B. jap._18hpi_rep_3
Symbiont res._Williams 82_B. jap._18hpi_rep_4
Control
Symbiont res._Williams 82_con_18hpi_rep_1
Symbiont res._Williams 82_con_18hpi_rep_2
Symbiont res._Williams 82_con_18hpi_rep_3
Symbiont res._Williams 82_con_18hpi_rep_4
Experimental
Symbiont res._Williams 82_B. jap._24hpi_rep_1
Symbiont res._Williams 82_B. jap._24hpi_rep_2
Symbiont res._Williams 82_B. jap._24hpi_rep_3
Control
Symbiont res._Williams 82_con_24hpi_rep_1
Symbiont res._Williams 82_con_24hpi_rep_2
Symbiont res._Williams 82_con_24hpi_rep_3
Experimental
Symbiont res._Williams 82_B. jap._48hpi_rep_1
Symbiont res._Williams 82_B. jap._48hpi_rep_2
Control
Symbiont res._Williams 82_con_48hpi_rep_1
Symbiont res._Williams 82_con_48hpi_rep_2
0%
100%
Percent of Expression Potential
created with GENEVESTIGATOR

Supplementary Figure 8. Microarray analysis of Voltage gated K^+ channel family genes. **(A)** Genes expression across five developmental stages. **(B)** Genes expression in 68 anatomical parts. **(C)** Detailed view of genes expressed for *B. japnicum* inoculation to root hairs for nodulation in soybean.

Supplementary Figure 9.

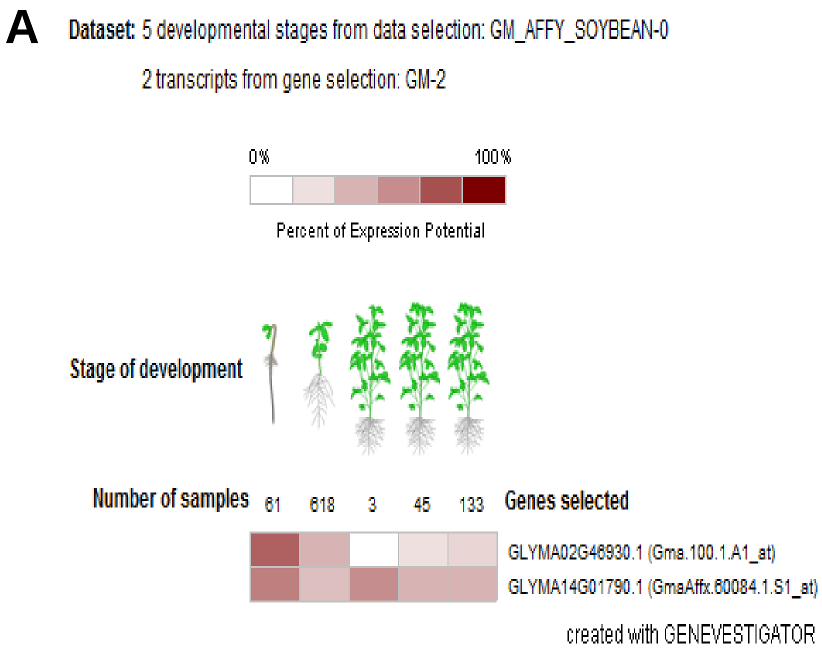

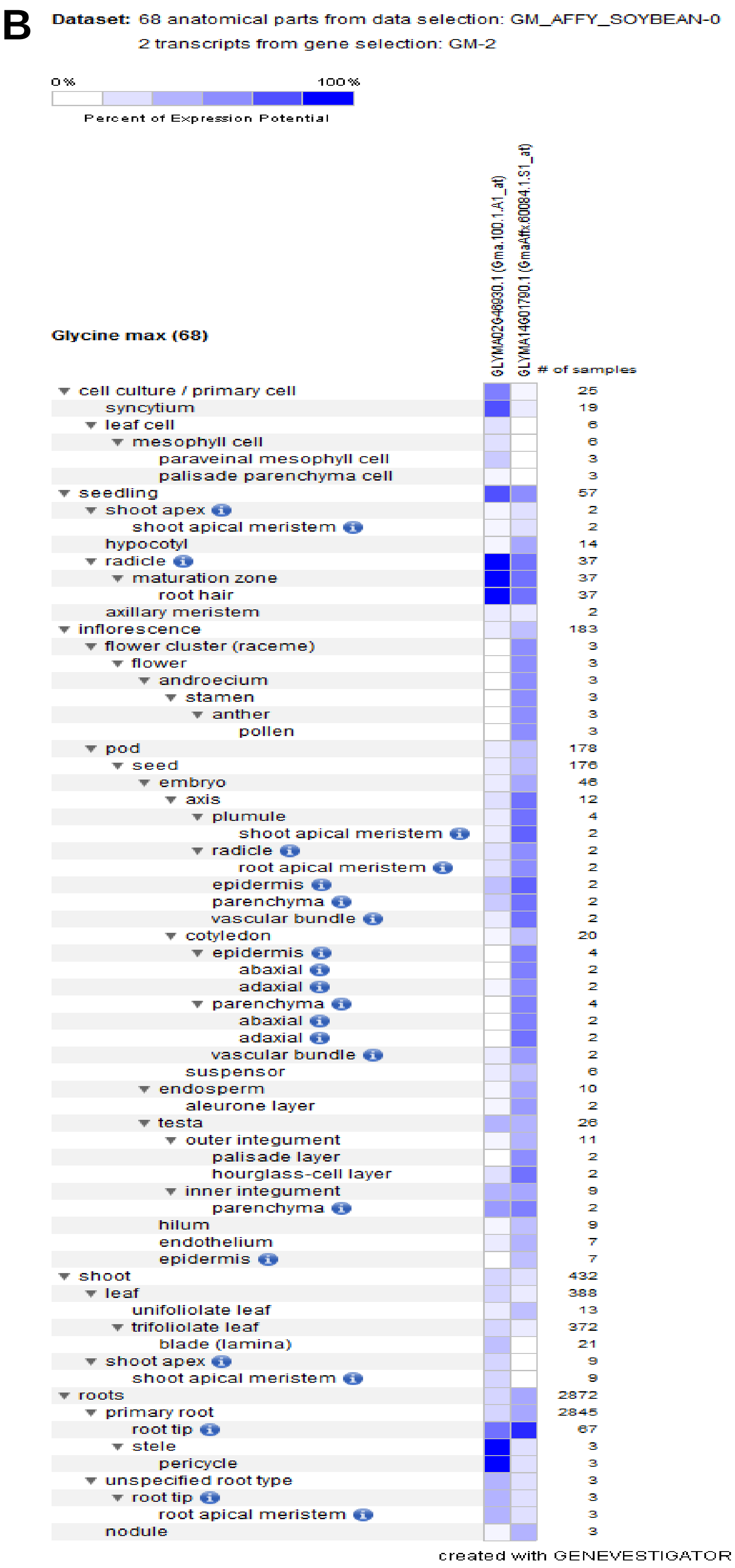
B
Dataset: 68 anatomical parts from data selection: GM_AFFY_SOYBEAN-0
2 transcripts from gene selection: GM-2
0%
100%
Percent of Expression Potential
Glycine max (68)
GLYMA02G48930.1 (Gma.100.1.A1_at)
GLYMA14G01780.1 (GmaAffx.60084.1.S1_at)
# of samples
cell culture / primary cell 25
syncytium 19
leaf cell 6
mesophyll cell 6
paraveinal mesophyll cell 3
palisade parenchyma cell 3
seedling 57
shoot apex 2
shoot apical meristem 2
hypocotyl 14
radicle 37
maturation zone 37
root hair 37
axillary meristem 2
inflorescence 183
flower cluster (raceme) 3
flower 3
androecium 3
stamen 3
anther 3
pollen 3
pod 178
seed 176
embryo 46
axis 12
plumule 4
shoot apical meristem 2
radicle 2
root apical meristem 2
epidermis 2
parenchyma 2
vascular bundle 2
cotyledon 20
epidermis 4
abaxial 2
adaxial 2
parenchyma 4
abaxial 2
adaxial 2
vascular bundle 2
suspensor 6
endosperm 10
aleurone layer 2
testa 26
outer integument 11
palisade layer 2
hourglass-cell layer 2
inner integument 9
parenchyma 2
hilum 9
endothelium 7
epidermis 7
shoot 432
leaf 388
unifoliolate leaf 13
trifoliolate leaf 372
blade (lamina) 21
shoot apex 9
shoot apical meristem 9
roots 2872
primary root 2845
root tip 67
stele 3
pericycle 3
unspecified root type 3
root tip 3
root apical meristem 3
nodule 3
created with GENEVESTIGATOR

C Detailed view of selected perturbations (absolute expression levels)

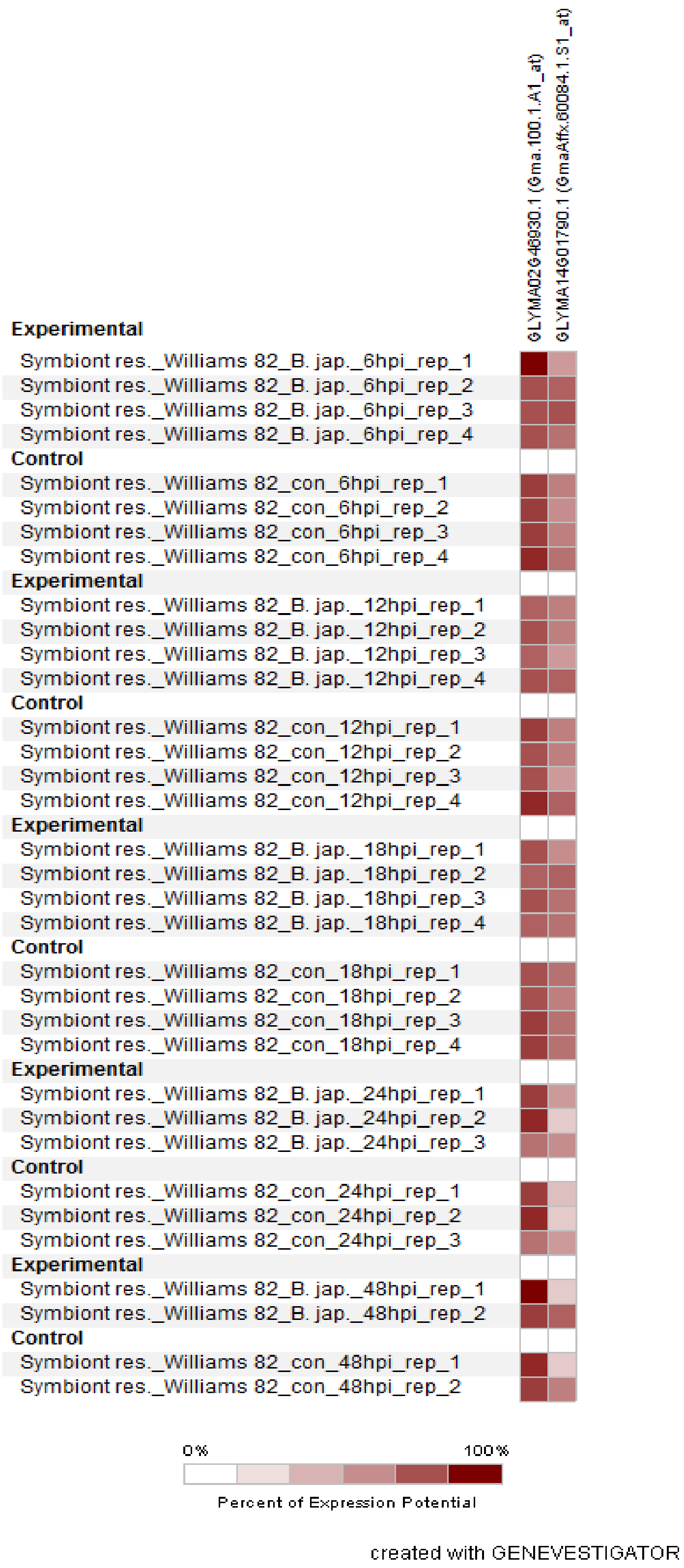

Supplementary Figure 9. Microarray analysis of TPK/KCO family genes. **(A)** Genes expression across five developmental stages. **(B)** Genes expression in 68 anatomical parts. **(C)** Detailed view of genes expressed for *B. japnicum* inoculation to root hairs for nodulation in soybean.

Supplementary Figure 10.

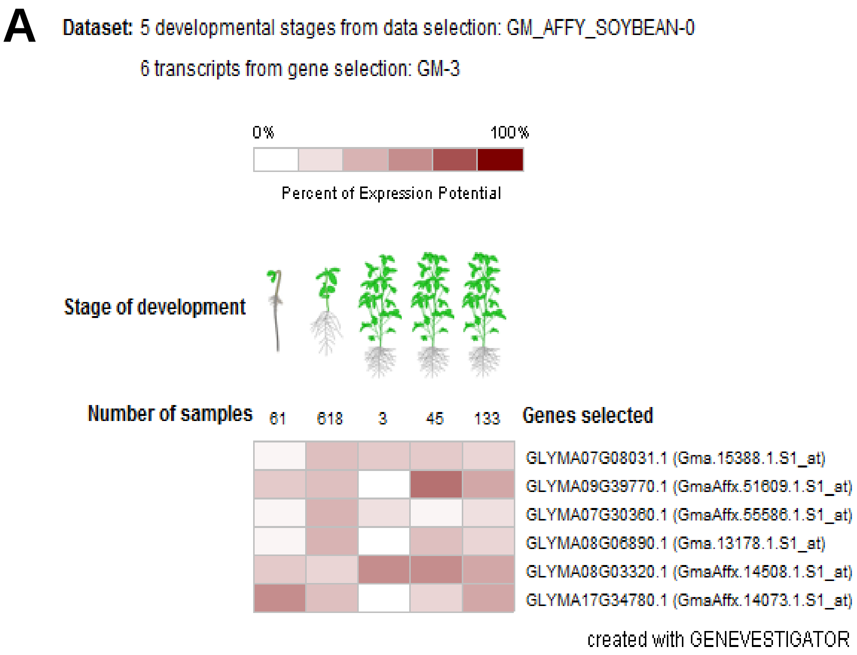

B

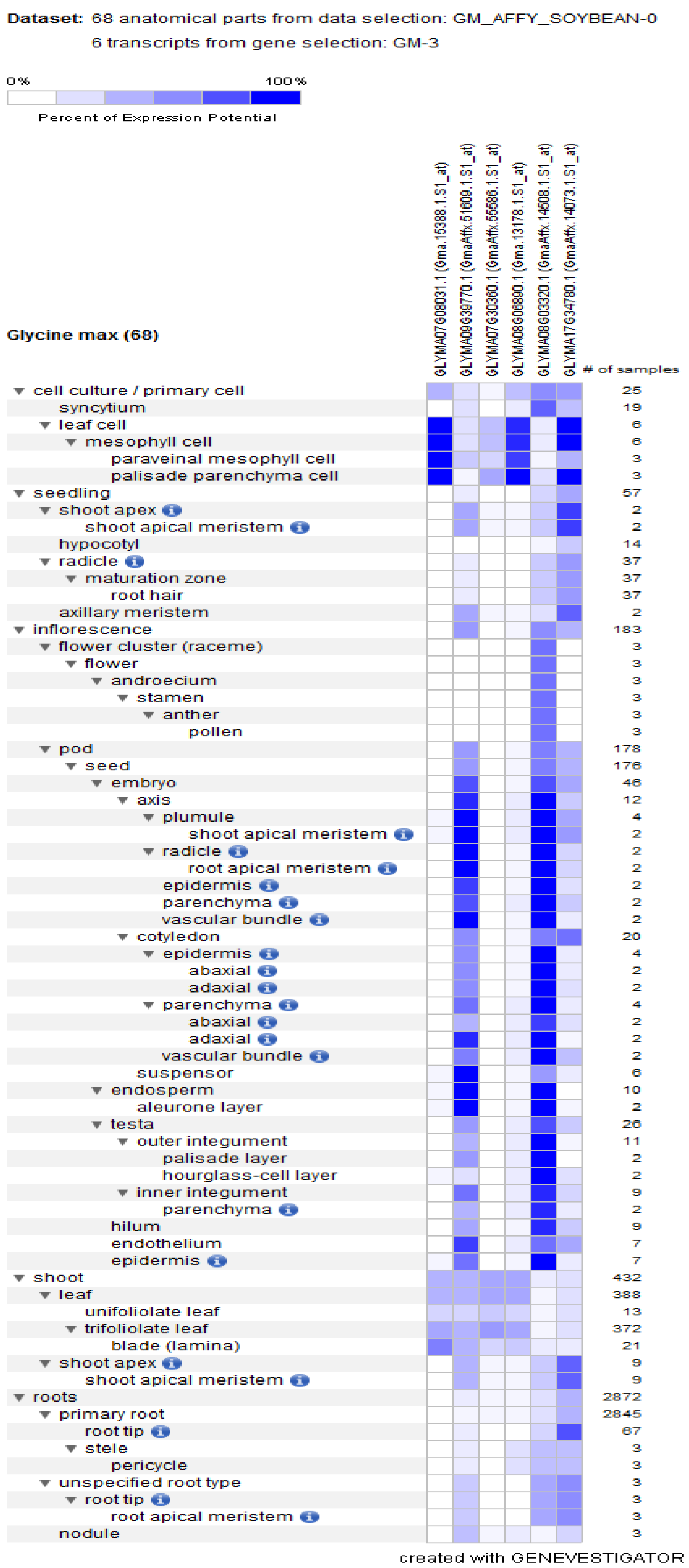
Dataset: 68 anatomical parts from data selection: GM_AFFY_SOYBEAN-0
6 transcripts from gene selection: GM-3
0%
100%
Percent of Expression Potential
Glycine max (68)
GLYMA07G08031.1 (Gma.15388.1.S1_at)
GLYMA09G39770.1 (GmaAffx.51809.1.S1_at)
GLYMA07G30380.1 (GmaAffx.55586.1.S1_at)
GLYMA08G06890.1 (Gma.13178.1.S1_at)
GLYMA08G03320.1 (GmaAffx.14608.1.S1_at)
GLYMA17G34780.1 (GmaAffx.14073.1.S1_at)
# of samples
cell culture / primary cell 25
syncytium 19
leaf cell 6
mesophyll cell 6
paraveinal mesophyll cell 3
palisade parenchyma cell 3
seedling 57
shoot apex 2
shoot apical meristem 2
hypocotyl 14
radicle 37
maturation zone 37
root hair 37
axillary meristem 2
inflorescence 183
flower cluster (raceme) 3
flower 3
androecium 3
stamen 3
anther 3
pollen 3
pod 178
seed 176
embryo 46
axis 12
plumule 4
shoot apical meristem 2
radicle 2
root apical meristem 2
epidermis 2
parenchyma 2
vascular bundle 2
cotyledon 20
epidermis 4
abaxial 2
adaxial 2
parenchyma 4
abaxial 2
adaxial 2
vascular bundle 2
suspensor 6
endosperm 10
aleurone layer 2
testa 26
outer integument 11
palisade layer 2
hourglass-cell layer 2
inner integument 9
parenchyma 2
hilum 9
endothelium 7
epidermis 7
shoot 432
leaf 388
unifoliolate leaf 13
trifoliolate leaf 372
blade (lamina) 21
shoot apex 9
shoot apical meristem 9
roots 2872
primary root 2845
root tip 67
stele 3
pericycle 3
unspecified root type 3
root tip 3
root apical meristem 3
nodule 3
created with GENEVESTIGATOR

C

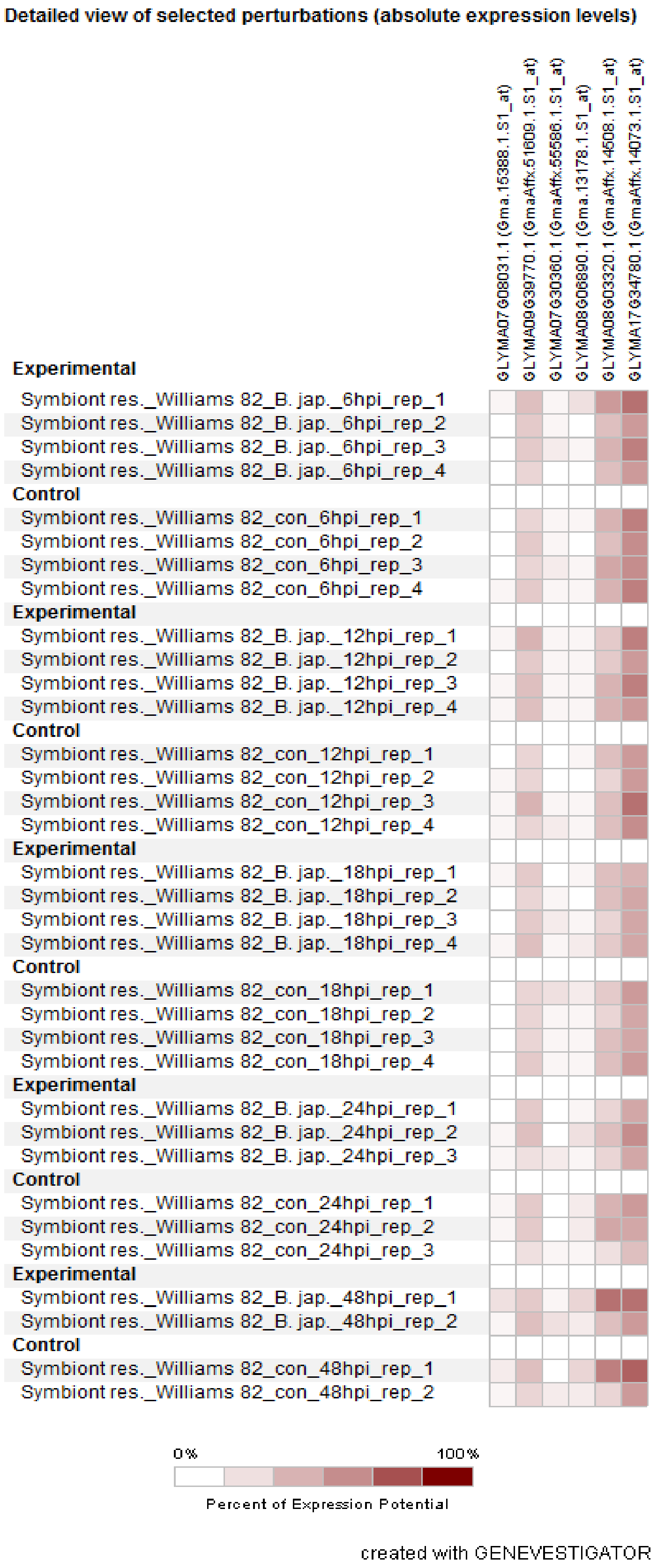
Detailed view of selected perturbations (absolute expression levels)
GLYMA07G08031.1 (Gma.15388.1.S1_at)
GLYMA09G39770.1 (GmaAffx.51609.1.S1_at)
GLYMA07G30360.1 (GmaAffx.55586.1.S1_at)
GLYMA08G06890.1 (Gma.13178.1.S1_at)
GLYMA08G03320.1 (GmaAffx.14508.1.S1_at)
GLYMA17G34780.1 (GmaAffx.14073.1.S1_at)
Experimental
Symbiont res._Williams 82_B. jap._6hpi_rep_1
Symbiont res._Williams 82_B. jap._6hpi_rep_2
Symbiont res._Williams 82_B. jap._6hpi_rep_3
Symbiont res._Williams 82_B. jap._6hpi_rep_4
Control
Symbiont res._Williams 82_con_6hpi_rep_1
Symbiont res._Williams 82_con_6hpi_rep_2
Symbiont res._Williams 82_con_6hpi_rep_3
Symbiont res._Williams 82_con_6hpi_rep_4
Experimental
Symbiont res._Williams 82_B. jap._12hpi_rep_1
Symbiont res._Williams 82_B. jap._12hpi_rep_2
Symbiont res._Williams 82_B. jap._12hpi_rep_3
Symbiont res._Williams 82_B. jap._12hpi_rep_4
Control
Symbiont res._Williams 82_con_12hpi_rep_1
Symbiont res._Williams 82_con_12hpi_rep_2
Symbiont res._Williams 82_con_12hpi_rep_3
Symbiont res._Williams 82_con_12hpi_rep_4
Experimental
Symbiont res._Williams 82_B. jap._18hpi_rep_1
Symbiont res._Williams 82_B. jap._18hpi_rep_2
Symbiont res._Williams 82_B. jap._18hpi_rep_3
Symbiont res._Williams 82_B. jap._18hpi_rep_4
Control
Symbiont res._Williams 82_con_18hpi_rep_1
Symbiont res._Williams 82_con_18hpi_rep_2
Symbiont res._Williams 82_con_18hpi_rep_3
Symbiont res._Williams 82_con_18hpi_rep_4
Experimental
Symbiont res._Williams 82_B. jap._24hpi_rep_1
Symbiont res._Williams 82_B. jap._24hpi_rep_2
Symbiont res._Williams 82_B. jap._24hpi_rep_3
Control
Symbiont res._Williams 82_con_24hpi_rep_1
Symbiont res._Williams 82_con_24hpi_rep_2
Symbiont res._Williams 82_con_24hpi_rep_3
Experimental
Symbiont res._Williams 82_B. jap._48hpi_rep_1
Symbiont res._Williams 82_B. jap._48hpi_rep_2
Control
Symbiont res._Williams 82_con_48hpi_rep_1
Symbiont res._Williams 82_con_48hpi_rep_2
0%
100%
Percent of Expression Potential
created with GENEVESTIGATOR

Supplementary Figure 10. Microarray analysis of KEA family genes. **(A)** Genes expression across five developmental stages. **(B)** Genes expression in 68 anatomical parts. **(C)** Detailed view of genes expressed for *B. japnicum* inoculation to root hairs for nodulation in soybean.
